# Oleic-Acid-Derived Nonionic Amphiphiles as Nanoscale Drug Delivery Vehicles

**DOI:** 10.3390/pharmaceutics18101209

**Published:** 2026-09-23

**Authors:** Sudhanshu Vats, Aarti, Natalie Hanheiser, Mathias Dimde, Abhishek K. Singh, Sunil K. Sharma

**Affiliations:** 1 Department of Chemistry, University of Delhi, Delhi 110007, India; sudhanshuvats98@gmail.com (S.V.); itsaartiyadav@gmail.com (A.); 2 Pt. Nekiram Sharma Government College Rohtak, Department of Higher Education, Haryana 124001, India; 3 Institut für Chemie und Biochemie, Freie Universität Berlin, Takustraße 3, 14195 Berlin, Germany; natalieah99@zedat.fu-berlin.de (N.H.); matze111@zedat.fu-berlin.de (M.D.); 4 Forschungszentrum für Elektronenmikroskopie, Core-Facility BioSupraMol, Institute of Chemistry and Biochemistry, Freie Universität Berlin, Fabeckstraße 36a, 14195 Berlin, Germany; 5 School of Biosciences, Chanakya University, Bengaluru 562165, India

**Keywords:** oleic acid, nonionic amphiphiles, self-assembly, nanocarriers, guest encapsulation, enzymatic release

## Abstract

**Background/Objectives:** The rational design of amphiphilic molecules that form stable nanostructures in aqueous media has attracted considerable attention for biomedical and drug-delivery applications. In this study, we designed and synthesized oleic-acid-based non-ionic amphiphiles incorporating different hydrophobic chains (alkyl/fluoroalkyl) and hydrophilic units such as polyethylene glycol (PEG) and lactose azide. **Methods:** The aggregation behavior and size distribution of the amphiphiles were investigated using dynamic light scattering (DLS), while the morphology of the self-assembled nanostructures was confirmed by transmission electron microscopy (TEM). The critical aggregation concentration (CAC) was determined using Nile red as a fluorescent probe. Nile red, nimodipine, and curcumin were used as hydrophobic cargo to assess amphiphile transport potential, while L929 fibroblast cell lines were used for cytotoxicity tests. Using Novozym 435, stimuli-responsive guest release was investigated. **Results:** All amphiphiles self-assembled into well-defined nanoscale micellar structures in aqueous medium, as shown by DLS and TEM. CAC ranged from 10^−4^ to 10^−5^ M. Cytotoxicity assessments demonstrated that none of the amphiphiles exhibited measurable toxicity at concentrations up to 0.5 mg/mL, affirming their biocompatibility. Alkylated amphiphiles displayed superior encapsulation efficiency for hydrophobic dye and drugs. The fluorescence intensity dropped 100% within 66 h in enzyme-triggered cargo release experiments, indicating regulated cargo release. **Conclusions:** Collectively, these findings demonstrate the robust encapsulation capacity and stimuli-responsive release behavior of these amphiphilic nanoarchitectures, establishing them as promising platforms for the development of advanced nanocarriers in biomedical applications.

## 1. Introduction

The recent advances in drug design and development have led to renewed interest in allied areas of pharmaceutical sciences. The development of drug reservoirs and carriers is one among them due to its enormous biomedical applications. The drug carriers have been immensely explored in the past two decades for the encapsulation and prolonged circulation of drugs and dyes and their controlled release at the target site. Since the properties of carriers can be tailored and modified by varying the constituents, this provides the opportunity to develop customized delivery vehicles for different drugs/biomolecules.

The literature search revealed that amphiphilic systems have drawn a lot of attention to address the issues associated with drugs having limited aqueous solubility and low bioavailability [1,2,3]. Amphiphilic systems proved to be flexible and smart excipients in the ongoing search for better permeation, bioavailability, and site-specific delivery across different administration routes.

Composed of covalently linked hydrophilic and hydrophobic segments, amphiphiles spontaneously self-assemble in aqueous environments into well-defined nanostructures, including micelles, vesicles, and other supramolecular aggregates, whose morphology and physicochemical properties are dictated by their molecular architecture [4,5].

Furthermore, the emerging research focuses involve developing smart nanocarriers that can exhibit stimuli-responsive behavior enabling the drug release in a controlled manner [6]. The stimuli can either be internal e.g., endolysosomal pH, redox potential, enzymatic activities, and monosaccharide concentration, or external, like physical stimuli such as temperature, light, electric field, mechanical force, and ultrasound. The nano-transporters can be made stimuli-responsive by conjugating an appropriate functional group.

The properties of amphiphilic systems are known to be significantly influenced by the selection of hydrophobic building blocks. In this regard, oleic acid (OA), a naturally occurring mono-unsaturated fatty acid, has become a particularly appealing bio-derived component. Its exceptional biocompatibility, biodegradability, structural flexibility resulting from the *cis*-configured olefinic bond, and fundamental membrane-interacting ability collectively facilitate enhanced drug encapsulation and cellular permeation [7,8]. OA-based amphiphiles also show favorable fluidity and packing properties, producing stable, water-soluble nanoaggregates with a high drug-loading capacity. In addition to its structural role, oleic acid has been demonstrated to have intrinsic therapeutic potential [9,10]. In order to show that OA actively promotes intracellular delivery in addition to driving self-assembly, Zhang et al. developed an OA-grafted copolymeric nano-micellar system, demonstrating high encapsulation efficiency, excellent biocompatibility, enhanced cellular uptake, and stimuli-triggered cargo release [11]. The multifunctional potential of OA-based amphiphilic assemblies was further demonstrated by Rinaldi et al. They reported OA-based nano-emulsions and niosomes for melanoma treatment, where OA contributed to both nanocarrier formation and antimetastatic activity. These systems revealed good physicochemical stability, efficient cellular uptake, and negligible toxicity toward normal cells collectively [12].

To tailor the water solubility, biocompatibility, and targeting ability of amphiphilic nanocarriers, selecting appropriate hydrophilic segments is equally important [13]. In this endeavor, PEG has been widely used as it is biocompatible, immunogenic, and able to prolong drug circulation [14]. Carbohydrate-based units, on the other hand, have the added benefits of being biodegradable and allowing receptor-mediated active targeting through lectin–carbohydrate interactions [15]. The incorporation of stimuli-responsive linkages to the amphiphilic framework adds another level of upgradation by enabling cargo to be released under relevant conditions [16]. Ester bonds have drawn significant interest as enzymatically cleavable motifs that experience selective hydrolysis in the presence of esterases overexpressed in tumor microenvironments, facilitating controlled and site-specific release of encapsulated drugs [17]. The controlled drug release is also influenced by the nature of the hydrophobic segment, e.g., fluoroalkyl and perfluoroalkyl chains exhibit remarkable hydrophobic and lipophobic properties due to the stability of C-F bond and their elevated surface activity [18,19,20,21]. These studies demonstrate that fine-tuning the hydrophobic–hydrophilic balance and integrating enzyme-responsive linkages are essential for effective self-assembly, substantial drug loading, and regulated release.

Amphiphiles consisting of oleic acid, alkyl/fluoroalkyl chains, and PEG/carbohydrate headgroups are known to possess low CAC, high stability, good biocompatibility, and better drug delivery performance [22]. Inspired by this study, we herein present the design, synthesis, and systemic characterization of six oleic-acid-based amphiphiles which exhibit stimuli-responsiveness toward lipase. The six oleic-acid-derived amphiphiles were designed to systematically vary the hydrophobic and hydrophilic groups while retaining a common lipase-cleavable ester linkage. These structural variations were introduced to tune the hydrophilic–lipophilic balance and self-assembly behavior and to evaluate their influence on the encapsulation and transport of hydrophobic guest molecules. These amphiphiles have been synthesized by incorporating mPEG and carbohydrate units as hydrophilic segments, and alkyl/fluoroalkyl chains as hydrophobic moieties. The synthesized amphiphiles form nano-sized aggregates in aqueous medium. Their self-assembly behavior and aggregate morphology were examined by fluorescence and UV-Vis spectroscopy and employing dynamic light scattering; Nile red was used as a model fluorescent dye. Nimodipine and curcumin were used as hydrophobic guests to study the encapsulation and transport potential of the resulting nano-assemblies. In vitro enzyme-triggered release of the encapsulated cargo was investigated using immobilized *Candida antarctica* lipase (Novozym 435). The biocompatibility of the amphiphiles was assessed through cytotoxicity studies. The relationship between molecular architecture, self-assembly behavior, encapsulation efficiency, triggered release, and biological performance is systematically discussed herein.

## 2. Materials and Methods

Chemicals and solvents from Sigma-Aldrich Chemicals, St. Louis, MO, USA and Spectrochem (SRL) Pvt. Ltd., Mumbai, Maharashtra, India were employed, with solvents distilled prior to use. Immobilized *Candida antarctica* lipase (Novozym 435) was obtained from Julich Chiral Solution GmbH (Jülich, Germany). Dyes and drugs for encapsulation studies were sourced from Sigma-Aldrich Chemicals, St. Louis, MO, USA and Fluka Chemie GmbH (Buchs, Switzerland), ensuring high purity. Merck silica gel 60F_254_ (Merck Group, Darmstadt, Germany) pre-coated TLC plates were utilized for reaction monitoring, while silica gel (100–200 mesh size) facilitated column chromatography. Visualizing TLC spots involved using Ceric and KMnO_4_ stains after gentle heating. Milli-Q water, acquired from Merck Millipore Milli-Q Internal System, was used for sample preparation for physicochemical characterization and transport analysis.

### 2.1. Critical Aggregation Concentration (CAC) Measurements

The critical aggregation concentration (CAC) of the synthesized amphiphiles was determined using fluorescence measurement with Nile red as a probe [23]. Initially a stock solution of Nile red dye was prepared at a concentration of 1 mg/mL in HPLC grade THF. Subsequently, 20 µL of this stock solution was added to each of 10 empty vials, and THF solvent was allowed to evaporate, resulting in the formation of a thin film of Nile red. Meanwhile the amphiphilic stock solution of each amphiphile was prepared at a concentration of 1 mM using Milli-Q water and stirred for 1 h. Serial dilution of the stock solution was performed to obtain different concentration of the amphiphiles, which were then transferred to the vials containing the thin film of Nile red and left to stir overnight. The unencapsulated dye in all solutions was removed using a polytetrafluoroethylene (PTFE) micro-filter (0.45 µm), and fluorescence measurements were conducted using a Cary-Eclipse fluorescence spectrophotometer (Agilent Technologies, Santa Clara, CA, USA). The fluorescence intensity maxima values (λ: ~620–640 nm) were plotted against the logarithm of the amphiphilic concentration for different samples to determine the CAC values.

### 2.2. Dynamic Light Scattering (DLS) Measurements

The characterization of nanostructure formed in the aqueous solution at a concentration of 5 mg/mL, well above their CACs, was conducted using a Malvern Zetasizer Nano ZS analyzer (Malvern Panalytical, Malvern, UK). The analyzer featured a thermostated sample chamber equipped with a 4 mW He-Ne laser and utilized backscattering detection (Scattering angle θ = 173°) with an avalanche photodiode as a detector. Disposable micro–BRAND UV-Cuvettes were employed for the measurements. After constant stirring for 24 h, the samples underwent filtration using a 0.45 µm PTFE micro filter, followed by the DLS measurements. Each measurement was performed in triplicate, with 10 runs per single measurement, and the mean values were calculated and utilized.

### 2.3. Transmission Electron Microscopy (TEM)

For TEM measurement, droplets of the sample solution (5 μL) were applied to hydrophilized Formvar^®^ S162-4 Plano Plano GmbH, Ernst-Befort-Strasse 12, D-35578, Wetzlar, Germany-supported carbon-coated copper grids (400 mesh) for 60 s and stained with uranyl acetate solution for 45 s (negative stain). Hydrophilization was achieved beforehand by 60 s glow discharging in an Emscope SC 500A device (Emscope SC500A, Emscope, Ashford, UK) at 20 mA. The supernatant fluid was removed by blotting with a filter paper. Measurements were carried out using a TALOS L120C electron microscope (Thermo Fisher Scientific Inc., Waltham, MA, USA). The microscope was equipped with a LaB6 cathode and operated at 120 kV accelerating voltage. Micrographs were acquired on a FEI Ceta CMOS camera (Thermo Fisher Scientific Inc., Waltham, MA, USA).

The diameter of the micelles was determined with ImageJ 1.53k. For the worm-like micelles, the diameter of the top views was measured. 

### 2.4. Drug/Dye Encapsulation and Quantification

Encapsulation of Nile red, nimodipine, and curcumin was achieved using the thin film method [24]. Initially, an aqueous stock solution of the amphiphiles at a concentration of 5 mg/mL and THF stock solutions of model guests Nile red (1 mg/mL)/curcumin (1 mg/mL)/nimodipine (4 mg/mL) were prepared. Aliquots of respective guest solutions, i.e., Nile red (120 µL)/curcumin (500 µL)/nimodipine (400 µL), were transferred to individual sample vials, and the THF was allowed to evaporate for 12 h to form a uniform layer of dye/drug at the vial’s bottom. Subsequently, 1 mL of an aqueous solution of the amphiphiles was added to each vial. After stirring the sample solutions at room temperature for 24 h, any un-encapsulated dye/drug was removed using a 0.45 µm PTFE filter, and both fluorescence and UV-Vis measurements were conducted. To quantify the encapsulated dye or drug, 200 µL of the stock solution of the encapsulated samples were lyophilized and re-dissolved in a known volume of anhydrous methanol (for Nile red and curcumin) or anhydrous ethanol (for Nimodipine). UV-Vis absorbance (200–800 nm) was recorded using a Cary-300 series UV-Vis (Agilent Technologies, Santa Clara, CA, USA) spectrophotometer, while fluorescence measurements (450–800 nm) were performed using Cary Eclipse (Agilent Technologies, Santa Clara, CA, USA) fluorescence spectrophotometer with a slit width of 5.0 nm and excitation wavelength 550 nm for Nile red and 420 nm for curcumin. The transport potential and wt.% encapsulation of all amphiphiles encapsulated with Nile red, nimodipine, and curcumin were determined by applying the Beer–Lambert law (molar extinction coefficient = 45,000 M^−1^ cm^−1^ at 552 nm for Nile red, 7200 M^−1^ cm^−1^ at 365 nm for nimodipine, and 55,306 M^−1^ cm^−1^ at 420 nm for curcumin). The amount of non-encapsulated drug was obtained directly by mass balance (initial drug added minus encapsulated drug quantified in the filtrate). These measurements were performed as single determinations. Data analysis was carried out using OriginPro 8 software.

### 2.5. Cytotoxicity Assay

All experiments were conducted following the German genetic engineering laws and German biosafety guidelines in the laboratory (S2). To ascertain the effect of amphiphiles on the viability of L929 cells, colorimetric assay using a water-soluble tetrazolium salt (WST-8) Cell Counting Kit 8 (CCK-8) assay was performed. L929 cells were cultivated in Dulbecco’s Modified Eagle’s medium (DMEM) supplemented with 10% fetal bovine serum (FBS) (Sigma-Aldrich, Co., Cat. No.: F7524) + 1% penicillin-streptomycin (PS) (Sigma-Aldrich, Co., Cat. No.: P4333) in the humidified atmosphere in 5% CO_2_ incubator at 37 °C. For cell viability measurements, the cells were plated in 96 well plates at a density of 2 × 10^4^ cells/mL and incubated overnight at 37 °C in CO_2_ incubator. Thereafter the cells were treated with amphiphiles with different concentrations (final concentrations are 0.015625, 0.03125, 0.0625, 0.125, 0.25, 0.5 mg mL^−1^) and further incubated for 24 h in CO_2_ incubator. Three hours prior to the termination, the cultured medium was removed from the wells and replaced with 180 µL of fresh medium and 20 µL of CCK-8 solution. The cells were again incubated for three hours in a dark humidified chamber at 37 °C in CO_2_ incubator. After incubation, the absorbance was measured (450 nm/650 nm) using a Tecan SPARK Plate Reader (Tecan Group Ltd., Zürich, Switzerland). The percentage viability was calculated as follows: All measurements were performed with three technical and one biological repeat. The cell viability was calculated by setting the negative control to 100% using the Excel software (Microsoft Excel 2024). All Graphs were plotted using GraphPad Prism 6.

Percent viability = OD value of treated sample/OD value of control sample × 100(1)

### 2.6. Enzyme Triggered Release Study

The enzyme-responsive behavior of the synthesized amphiphiles was investigated using fluorescence measurements, employing the dye Nile red as a model probe. Encapsulation of the dye was conducted by methods similar to those used for quantification. After eliminating the unencapsulated dye with a 0.45 µm PTFE filter, a few drops of *n*-butanol and 200 wt.% of immobilized *Candida antarctica* lipase (Novozym-435) were introduced. Subsequently, the final solutions were incubated at 37 °C and 200 rpm under dark. The release of the dye over time was monitored by periodically taking aliquots from the reaction mixture and measuring their fluorescence.

### 2.7. Critical Aggregation Determination Using Count Rate Method by DLS

The critical aggregation concentration (CAC) of the amphiphiles was conducted by the dynamic light scattering (DLS) count-rate method using a Malvern Zetasizer Nano ZS analyzer. The analyzer featured a thermostated sample chamber equipped with a 4 mW He-Ne laser and utilized backscattering detection (Scattering angle θ = 173°) with an avalanche photodiode as a detector. Disposable micro–BRAND UV-Cuvettes were employed for the measurements. Particle size was determined by the dynamic light scattering (DLS) count-rate method using a Malvern Zetasizer Nano ZS (Malvern Instruments, Malvern, UK). A stock solution of the amphiphile was prepared at a concentration of 2 mM in Mili-Q water. A series of ten amphiphile solutions of progressively decreasing concentration was prepared by serial two-fold dilution. Briefly, 1.0 mL of the amphiphile solution was mixed with 1.0 mL of Mili-Q water at each dilution step, and the procedure was continued sequentially to obtain the required concentration range. The count rate of each solution was measured under identical DLS measurement conditions. The measured count rate, expressed in kilo counts per second (kcps), was plotted against the logarithm of the amphiphile concentration [log(C)]. The CAC was determined from the concentration corresponding to the intersection of the two linear regions observed in the count-rate versus log concentration plot, representing the concentration-dependent change associated with aggregate formation.

### 2.8. Synthetic Procedure

Non-ionic mPEG and oleic-acid-based amphiphiles were synthesized via Cu(I) catalyzed Huisgen 1,3-dipolar cyclo-addition reaction (Scheme 1c). The hydrophobic and hydrophilic parts, in turn, were synthesized following Scheme 1a,b.

#### 2.8.1. Synthesis of ethyl (Z)-octadec-9-enoate (**2**)

Compound **1** (5.0 g, 1 equiv., 17.7 mmol) was dissolved in ethanol (60 mL), and concentrated H_2_SO_4_ was added in catalytic amount, and the reaction mixture was refluxed for 12 h. Progress of the reaction was monitored by TLC using ethyl acetate/petroleum ether (1:19). On completion of the reaction, the reaction mixture was concentrated under reduced pressure and the obtained crude product was suspended in water (50 mL) and extracted with chloroform (3 × 50 mL). The combined organic layer was dried over anhydrous Na_2_SO_4_ and the solvent evaporated to yield the crude product, which was purified through eluting column chromatography using ethyl acetate/petroleum ether to give the desired compound **2** as a transparent liquid in 77% yield.

**IR** (**KBr**) **ν_max_**: 2921, 2856, 1737 (C=O), 1455 (-C=C-), 1377, 1240, 1030, 722 cm^−1^; **^1^H NMR** (**400 MHz**, **CDCl_3_**): δ 0.88 (t, 3H, *^3^J* = 7.2 Hz, H-18), 1.23–1.30 (m, 23H, H-4–H-7, H-12–H-17 & H-b), 1.58–1.67 (m, 2H, H-3), 1.98–2.05 (m, 4H, H-8 & H-11), 2.28 (t, 2H, *^3^J* = 7.6 Hz, H-2), 4.12 (q, 2H, *^3^J* = 6.8 Hz, H-a), 5.30–5.38 (m, 2H, H-9 & H-10); **^13^C NMR** (**100.5 MHz**, **CDCl_3_**): δ 14.17 (C-18), 14.31 (C-b), 22.75, 25.04, 27.22, 27.28, 29.16, 29.18, 29.23, 29.39, 29.59, 29.75, 29.83, 31.97, 34.44 (C-2-C-8 & C-11-C-17), 60.21 (C-a), 129.81 (C-9), 130.05 (C-10), 173.96 (C-1); HRMS: *m*/*z* [M+H]^+^ Calculated for C_20_H_38_O_2_: 310.2872; found: 310.2871.

#### 2.8.2. Synthesis of ethyl 8-(3-octyloxiran-2-yl)octanoate (**3**)

To a stirring solution of compound **2** (2 g, 1 equiv., 6.0 mmol) in DCM (40 mL), *m*-CPBA (2.22, 2 equiv., 12 mmol) was added at 0 °C and the reaction mixture was stirred for 4 h. Progress of the reaction was monitored by TLC using ethyl acetate/petroleum ether (1:19). On completion of the reaction, the reaction mixture was suspended in saturated solution of NaHCO_3_ (to remove *m*-chlorobenzoic acid) and extracted with chloroform (4 × 30 mL). The combined organic layer was dried over anhydrous Na_2_SO_4_ and the solvent evaporated to yield the crude product, which was purified through eluting column chromatography using petroleum ether/ethyl acetate to give the desired compound **3** as a transparent liquid in 95% yield.

**IR** (**KBr**) **ν_max_**: 2921, 2849, 1737 (C=O), 1383, 1246, 1023, 727 cm^−1^; **^1^H NMR** (**400 MHz**, **CDCl_3_**): δ 0.88 (t, 3H, *^3^J* = 7.2 Hz, H-18), 1.23–1.33 (m, 23H, H-4–H-7, H-12–H-17 & H-b), 1.45–1.49 (m, 4H, H-8 & H-11), 1.58–1.64 (m, 2H, H-3), 2.28 (t, 2H, *^3^J* = 7.6 Hz, H-2), 2.89–2.92 (m, 2H, H-9 & H-10), 4.12 (q, 2H, *^3^J* = 6.8 Hz, H-a); **^13^C NMR** (**100.5 MHz**, **CDCl_3_**): δ 14.07 (C-18), 14.23 (C-b), 22.64, 24.90 26.54, 26.59, 27.78, 27.81, 29.01, 29.17, 29.21, 29.32, 29.51, 29.53, 31.84, 34.31 (C-2-C-8 & C-11-C-17), 57.16 (C-9), 57.21 (C-10), 60.13 (C-a), 173.81 (C-1); HRMS: *m*/*z* [M+H]^+^ Calculated for C_20_H_38_O_3_: 326.282; found: 326.2821.

#### 2.8.3. Synthesis of ethyl 9,10-dihydroxyoctadecanoate (**4**)

To a solution of compound **3** (1.5 g, 1 equiv., 4.0 mmol) in THF:H_2_O (1:1) (40 mL), 0.5 mL of conc. H_2_SO_4_ was added and the reaction mixture was stirred at room temperature for 12 h. Progress of the reaction was monitored by TLC using ethyl acetate/petroleum ether (3:7). To quench the reaction, NaHCO_3_ was added and the reaction mixture was concentrated under reduced pressure. The obtained crude product was suspended in water and extracted with ethyl acetate (4 × 50 mL). The combined organic layer was dried over Na_2_SO_4_ and the solvent evaporated to yield the crude product, which was purified through column chromatography using petroleum ether/ethyl acetate to give the desired compound as a white solid in 63% yield.

**IR** (**KBr**) **ν_max_**: 3340 (-OH), 2915, 2843, 1740 (C=O), 1461, 1167, 1049, 723 cm^−1^; **^1^H NMR** (**400 MHz**, **CDCl_3_**): δ 0.85 (t, 3H, *^3^J* = 7.2 Hz, H-18), 1.21–1.38 (m, 23H, H-4–H-7, H-12–H-17 & H-b), 1.43–1.47 (m, 4H, H-8 & H-11), 1.55–1.62 (m, 2H, H-3), 2.26 (t, 2H, *^3^J* = 7.6 Hz, H-2), 3.34–3.39 (m, 2H, H-9 & H-10),4.09 (q, 2H, *^3^J* = 6.8 Hz, H-a); **^13^C NMR** (**100.5 MHz**, **CDCl_3_**): δ 14.19 (C-18), 14.32 (C-b), 22.74, 24.97, 25.64, 25.75, 29.10, 29.24, 29.36, 29.50, 29.64, 29.77, 31.95, 31.61, 33.67, 34.41 (C-2-C-8 & C-11-C-17), 60.31 (C-a), 74.54 (C-9), 74.60 (C-10), 174.07 (C-1); HRMS: *m*/*z* [M+H]^+^ Calculated for C_20_H_40_O_4_: 344.2926; found: 344.2927.

#### 2.8.4. Synthesis of 9,10-bis(prop-2-yn-1-yloxy)octadecanoic acid (**5**)

To a solution of NaH (0.27g, 8 equiv., 11 mmol) in DMF (30 mL), compound **4** (0.5 g, 1 equiv., 1 mmol) was added and stirred for 20 min under ice cold conditions to maintain the temperature at 0 °C in N_2_ atmosphere. Then, propargyl bromide (0.48 g, 2.5 equiv., 4mmol) was added and the reaction mixture was further stirred at room temperature for 12 h. Progress of the reaction was monitored by TLC using ethyl acetate/petroleum ether (3:7). On completion, the reaction mixture was concentrated under reduced pressure. The obtained crude product was suspended in water and extracted with chloroform (3 × 30 mL). The combined organic layer was dried over anhydrous Na_2_SO_4_ and the solvent evaporated to yield the crude product, which was purified through column chromatography using petroleum ether/ethyl acetate to give the desired compound as a yellow liquid in 72% yield.

**IR** (**KBr**) **ν_max_**: 3285 (≡*C*–*H*), 2951, 2846, 2155 (C≡C), 1685 (C=O), 1453, 1156, 1039, 735 cm^−1^; **^1^H NMR** (**400 MHz**, **CDCl_3_**): δ 0.88 (t, 3H, *^3^J* = 7.2 Hz, H-18), 1.27–1.41 (m, 20H, H-4–H-7 & H-12–H-17), 1.43–1.56 (m, 4H, H-8 & H-11), 1.59–1.64 (m, 2H, H-3), 2.34 (t, 2H, *^3^J* = 7.6 Hz, H-2), 2.41 (t, 2H, *^4^J* = 2.4 Hz, H-3′), 3.49–3.54 (m, 2H, H-9 & H-10), 4.25 (d, 4H, *^4^J* = 2.4 Hz, H-1′); **^13^C NMR** (**100.5 MHz**, **CDCl_3_**): δ 14.12 (C-18), 22.67, 24.64, 25.63, 25.72, 28.99, 29.14, 29.30, 29.53, 29.79, 29.83, 29.87, 31.88, 34.04 (C-2-C-8 & C-11-C-17), 57.75 (C-1’), 73.92 (C-3’), 79.97 (C-2’), 80.49 (C-9 & C-10), 180.01 (C-1); HRMS: *m*/*z* [M+H]^+^ Calculated for C_24_H_40_O_4_: 392.2934; found: 392.2927.

#### 2.8.5. Synthesis of hexadecyl 9,10-bis(prop-2-yn-1-yloxy)octadecenoate (**6**) and 3,3,4,4,5,5,6,6,7,7,8,8,8-tridecafluorooctyl 9,10-bis(prop-2-yn-1-yloxy)octdecanoate (**7**)

Compound **5** (0.2 g, 1 equiv., 0.5 mmol) and cetyl alcohol (0.12 g, 1 equiv., 0.4 mmol)/3,3,4,4,5,5,6,6,7,7,8,8,8-tridecafluorooctan-1-ol (0.18 g, 1 equiv., 0.4 mmol) were dissolved in DCM (30 mL). The mixture was stirred under ice cold conditions to maintain the temperature of 0 °C and EDC.HCl (0.2 g, 2 equiv., 1 mmol), and DMAP (0.06 g, 1 equiv., 0.5 mmol) were then added under inert atmosphere. After 15 min, the reaction mixture was allowed to stir at room temperature for 24 h. Progress of the reaction was monitored by TLC using ethyl acetate/petroleum ether (1:9). On completion of the reaction, the reaction mixture was concentrated under reduced pressure. The obtained crude product was suspended in water and extracted with chloroform (3 × 20 mL). The combined organic layer was dried over anhydrous Na_2_SO_4_ and the solvent evaporated to yield the crude product, which was purified through column chromatography using petroleum ether/ethyl acetate to give the desired compound **6**/**7**.

Compound **6**: Obtained as a transparent liquid in 75% yield by following the general procedure.

**IR** (**KBr**) **ν_max_**: 3286 (≡*C*–*H*), 2922, 2836, 2157 (C≡C), 1722 (C=O), 1453, 1231, 1126, 1089, 710 cm^−1^;**^1^H NMR** (**400 MHz**, **CDCl_3_**): δ 0.88 (t, 6H, *^3^J* = 7.2 Hz, H-18 & H-16”), 1.23–1.45 (m, 48H, H-4–H-7, H-12–H-17 & H-2”–H-15”), 1.51–1.64 (m, 6H, H-8, H-11 & H-3), 2.28 (t, 2H, *^3^J* = 7.6 Hz, H-2), 2.41 (t, 2H, *^4^J* = 2.4 Hz, H-3’), 3.48–3.55 (m, 2H, H-9 & H-10), 4.05 (t, 2H, *^3^J* = 6.8 Hz, H-1”), 4.25 (d, 4H, *^4^J* = 2.4 Hz, H-1’); **^13^C NMR** (**100.5 MHz**, **CDCl_3_**): δ 14.11 (C-18 & C-16”), 22.67, 22.69, 24.99, 25.66, 25.73, 25.93, 28.65, 29.12, 29.19, 29.30, 29.36, 29.53, 29.58, 29.65, 29.69, 29.79, 29.87, 31.88, 34.37 (C-2-C-8, C-11-C-17 & C-2”-C-15”), 57.75 (C-1”), 64.41 (C-1’), 73.87 (C-3’), 80.05 (C-2’), 80.51 (C-9 & C-10), 173.97 (C-1); HRMS: *m*/*z* [M+H]^+^ Calculated for C_40_H_72_O_4_: 616.5431; found: 616.5432.

Compound **7**: Obtained as a transparent liquid in 70% yield by following the general procedure.

**IR** (**KBr**) **ν_max_**: 3276 (≡*C*–*H*), 2920, 2850, 2167 (C≡C), 1725 (C=O), 1457, 1221, 1130, 1022, 704 cm^−1^; **^1^H NMR** (**400 MHz**, **CDCl_3_**): δ 0.86 (t, 3H, *^3^J* = 7.2 Hz, H-18), 1.24–1.62 (m, 26H, H-4–H-7, H-12–H-17, H-8, H-11 & H-3), 2.30 (t, 2H, *^3^J* = 7.6 Hz, H-2), 2.38 (t, 2H, *^4^J* = 2.4 Hz, H-3’), 2.40–2.51 (m, 2H, H-2”), 3.48–3.50 (m, 2H, H-9 & H-10), 4.23 (d, 4H, *^4^J* = 2.4 Hz, H-1’), 4.36 (t, 2H, *^3^J* = 6.8 Hz, H-1”); **^13^C NMR** (**100.5 MHz**, **CDCl_3_**): δ 14.14 (C-18), 22.72, 24.82, 25.73, 25.80, 29.10, 29.20, 29.59, 29.85, 29.96, 31.94, 34.11 (C-2-C-8, C-11-C-17 & C-2”), 57.82 (C-1’), 73.92 (C-1”), 80.09 (C-3’), 80.14 (C-2’), 80.58 (C-9 & C-10), 173.44 (C-1); **^19^F NMR** (**375 MHz**, **CDCl_3_**): δ −80.83 (F-8”), −113.52 (F-7”), −121.79 (F-6”), −122.78 (F-5”), −123.51 (F-4”), −126.05 (F-3”); HRMS: *m*/*z* [M+H]^+^ Calculated for C_32_H_43_F_13_O_4_: 738.6723; found: 738.6722.

#### 2.8.6. Synthesis of methoxypolyethylene glycol azide (**12**/**13**)

To a stirred and clear solution of methoxypolyethylene glycol (**8**/**9**) (M_n_: 550/750 gmol^−1^) (5.0 g, 1.0 equiv., 9 mmol and 6.6 mmol, respectively) and trimethyl amine (2.5 equiv.) in anhydrous DCM, mesyl chloride (1.5 equiv.) was added dropwise in ice cold condition and the solution stirred for 3 h. After completion of the reaction, the precipitate was removed by filtration and the mixture concentrated under vacuum to give mesylated product (**10**/**11**). The crude product (**10**/**11**) so obtained was then dissolved in anhydrous DMF (150 mL) and then sodium azide (2.5 equiv.) was added. The reaction mixture was stirred at 80 °C under N_2_ atmosphere for 16 h. After completion of the reaction, DMF was evaporated under reduced pressure and the product was extracted with chloroform (3× 100 mL). The crude compound (**12**/**13**) was purified by column chromatography using silica gel (100–200 mesh) (Chloroform:Methanol), and the compound (**12**/**13**) was obtained in 83% yield as viscous liquid. The obtained spectral data was compared with the literature value [25].

#### 2.8.7. General Procedure for the Synthesis of β-azido-D-lactose (**17**)

##### Synthesis of per-O-acetylated lactose (**15**)

A suspension of sodium acetate (1.19 g, 1.0 equiv., 14.5 mmol) in acetic anhydride (19.94 g, 13.3 equiv., 195.3 mmol) was refluxed for 20 min. D-lactose (**14**) (5 g, 1 equiv., 14.6 mmol) was then added in small lots and the mixture was further stirred for 30 min. After that, the reaction mixture was concentrated under reduced pressure and the obtained crude product was suspended in ice cold water (25 mL) and extracted with chloroform (3 × 30 mL). The combined organic layer was dried over anhydrous Na_2_SO_4_ and the solvent was evaporated to yield the crude product as white solid in 75% yield [26].

##### Synthesis of acetylated lactose azide (**16**)

To a cooled (0 °C) solution of per-*O*-acetylated lactose (4.7 g, 1 equiv., 6.9 mmol) in anhydrous DCM (35 mL), SnCl_4_ (0.9 g, 0.5 equiv., 3.4 mmol) was added dropwise under an inert atmosphere (N_2_ gas) and the solution was stirred for 15 min. TMSN_3_ (1.58, 2 equiv., 13.7 mmol) was then added dropwise and the reaction mixture was allowed to stir at 25 °C for 12 h. Progress of the reaction was monitored by TLC using ethyl acetate/petroleum ether (1:1). Upon completion, the reaction mixture was concentrated under reduced pressure, and the residue was suspended in water (15 mL) and extracted with chloroform (3 × 25 mL). The combined organic layers were dried over anhydrous Na_2_SO_4_ concentrated to yield the crude product, which was purified by column chromatography using ethyl acetate/petroleum ether to afford compound 16 as a white solid in 72% yield [26].

##### Synthesis of β-azido-D-lactose (**17**)

Sodium methoxide (1.0 M in methanol) was added to a solution of acetylated lactose azide (**16**) (2.0 g) in anhydrous methanol (50 mL) until pH reached 10 and the mixture was stirred at 25 °C for 12 h. The solution was then neutralized by acidic ion exchange resin (Dowex 50 W), filtered, and concentrated under reduced pressure to give the deprotected lactose azide (**17**) as white solid in 77% yield. The spectral data obtained was consistent with those reported in the literature [26,27].

#### 2.8.8. Synthesis of Compounds **18**–**21**

Hydrophobic (**6**/**7**) (0.1g, 1 equiv., 0.16 mmol and 0.13 mmol respectively) and hydrophilic moieties (**12**/**13**) (2.2 equiv.) were taken in round-bottom flask and dissolved in DMF (15 mL). Then bromotris(triphenylphosphine) copper(I) catalyst (5 mg) and DIPEA (2.5 equiv.) were added, and the reaction mixture was allowed to stir at 60 °C for 24 h. Progress of the reaction was monitored by TLC using methanol/chloroform (1:9). On completion of the reaction, DMF was removed in vacuo. Purification was done by column chromatography using silica gel (100–200 mesh) and 3% methanol in chloroform as eluent to yield the pure compound (**18**–**21**) in 80–85% yield.

Compound **18**: The click reaction of compound **7** with **12** gave compound **18** as a viscous liquid in 82% yield by following the general procedure.

**IR** (**KBr**) **ν_max_**: 2929, 2820, 1738 (C=O), 1415, 1173, 1023, 715 cm^−1^; **^1^H NMR** (**400 MHz**, **CDCl_3_**): δ 0.83 (t, 3H, *^3^J* = 6.8 Hz, H-18), 1.20–1.58 (m, 26H, H-3–H-8 & H-11–H-17), 2.27 (t, 2H, *^3^J* = 7.6 Hz, H-2), 2.37–2.49 (m, 2H, H-2”), 3.34 (s, 6H, H-31’), 3.50–3.61 (m, 90H, H-9’–H-30’, H-9 & H-10), 3.83 (t, 4H, *^3^J* = 5.2 Hz, H-7’), 4.33 (t, 2H, *^3^J* = 6.8 Hz, H-1”), 4.49 (t, 4H, *^3^J* = 5.2 Hz, H-8’), 4.60–4.73 (m, 4H, H-1’), 7.73 (s, 2H, H-3’); **^13^C NMR** (**100.5 MHz**, **CDCl_3_**): δ 14.17 (C-18), 22.72, 24.80, 25.85, 29.14, 29.39, 29.64, 29.91, 31.94, 34.08 (C-2-C-8, C-11-C-17 & C-2”), 50.20 (C-1”), 59.07 (C-7’), 64.18 (C-31’), 69.55 (C-1’), 70.51, 70.53, 70.58, 70.62, 71.96 (C-8’-C-30’), 80.74 (C-9 & C-10), 123.89 (C-3’), 145.54 (C-2’), 173.47 (C-1); **^19^F NMR** (**375 MHz**, **CDCl_3_**): δ −80.69 (F-8”), −113.54 (F-7”), −121.77 (F-6”), −122.79 (F-5”), −123.52 (F-4”), −126.05 (F-3”).

Compound **19**: The click reaction of compound **7** with **13** gave compound **19** as a viscous liquid in 84% yield by following the general procedure.

**IR** (**KBr**) **ν_max_**: 2960, 2826, 1728 (C=O), 1405, 1174, 1024, 716 cm^−1^; **^1^H NMR** (**400 MHz**, **CDCl_3_**): δ 0.82 (t, 3H, *^3^J* = 6.8 Hz, H-18), 1.18–1.57 (m, 26H, H-3–H-8 & H-11–H-17), 2.27 (t, 2H, *^3^J* = 7.6 Hz, H-2), 2.38–2.49 (m, 2H, H-2”), 3.33 (s, 6H, H-41’), 3.49–3.63 (m, 130H, H-9’–H-40’, H-9 & H-10), 3.83 (t, 4H, *^3^J* = 5.2 Hz, H-7’), 4.32 (t, 2H, *^3^J* = 6.8 Hz, H-1”), 4.48 (t, 4H, *^3^J* = 5.2 Hz, H-8’), 4.59–4.72 (m, 4H, H-1’), 7.72 (s, 2H, H-3’); **^13^C NMR** (**100.5 MHz**, **CDCl_3_**): δ 14.18 (C-18), 22.72, 24.79, 25.79, 29.13, 29.39, 29.64, 31.93, 34.07 (C-2–C-8, C-11–C-17 & C-2”), 50.19 (C-1”), 59.08 (C-7’), 64.20 (C-31’), 69.55 (C-1’), 70.54, 70.59, 70.64, 70.66, 71.96 (C-8’-C-30’), 80.71 (C-9 & C-10), 123.87 (C-3’), 145.52 (C-2’), 173.44 (C-1); **^19^F NMR** (**375 MHz**, **CDCl_3_**): δ −80.55 (F-8”), −113.39 (F-7”), −121.63 (F-6”), −122.65 (F-5”), −123.38 (F-4”), −125.91 (F-3”).

Compound **20**: The click reaction of compound **6** with **12** gave compound **20** as a viscous liquid in 85% yield by following the general procedure.

**IR** (**KBr**) **ν_max_**: 2963, 2844, 1729 (C=O), 1471, 1102, 1034, 719 cm^−1^; **^1^H NMR** (**400 MHz**, **CDCl_3_**): δ 0.84 (t, 6H, *^3^J* = 7.2 Hz, H-18 & H-16”), 1.21–1.57 (m, 54H, H-3–H-8, H-11–H-17 & H-2”–H-15”), 2.24 (t, 2H, *^3^J* = 7.6 Hz, H-2), 3.34 (s, 6H, H-31’), 3.50–3.62 (m, 90H, H-9’–H-30’, H-9 & H-10), 3.84 (t, 4H, *^3^J* = 4.8 Hz, H-7’), 4.01 (t, 2H, *^3^J* = 6.4 Hz, H-1”), 4.49 (t, 4H, *^3^J* = 4.8 Hz, H-8’), 4.60–4.73 (m, 4H, H-1’), 7.72 (s, 2H, H-3’); **^13^C NMR** (**100.5 MHz**, **CDCl_3_**): δ 14.19 (C-18 & C-16”), 22.74, 25.05, 25.82, 25.98, 28.71, 29.25, 29.33, 29.41, 29.59, 29.65, 29.71, 29.75, 29.92, 30.28, 30.74, 31.95, 31.98, 34.41 (C-2–C-8, C-11–C-17 & C-2”–C-15”), 50.20 (C-7’), 59.09 (C-31’), 64.23 (C-1”), 64.48 (C-1’), 69.57 (C-8’), 70.54, 70.56, 70.61, 70.65, 71.98 (C-9’–C-30’), 80.76 (C-9 & C-10), 123.84 (C-3’), 145.57 (C-2’), 174.04 (C-1).

Compound **21**: The click reaction of compound **6** with **13** gave compound **21** as a viscous liquid in 83% yield by following the general procedure.

**IR** (**KBr**) **ν_max_**: 2923, 2866, 1747 (C=O), 1465, 1192, 1034, 753 cm^−1^; **^1^H NMR** (**400 MHz**, **CDCl_3_**): δ 0.83 (t, 6H, *^3^J* = 7.2 Hz, H-18 & H-16”), 1.21–1.58 (m, 54H, H-3–H-8, H-11–H-17 & H-2”–H-15”), 2.24 (t, 2H, *^3^J* = 7.6 Hz, H-2), 3.33 (s, 6H, H-31’), 3.49–3.61 (m, 130H, H-9’–H-40’, H-9 & H-10), 3.83 (t, 4H, *^3^J* = 4.8 Hz, H-7’), 4.01 (t, 2H, *^3^J* = 6.4 Hz, H-1”), 4.49 (t, 4H, *^3^J* = 4.8 Hz, H-8’), 4.60–4.72 (m, 4H, H-1’), 7.72 (s, 2H, H-3’); **^13^C NMR** (**100.5 MHz**, **CDCl_3_**): δ 14.19 (C-18 & C-16”), 22.73, 25.04, 25.81, 25.97, 28.69, 29.31, 29.40, 29.58, 29.64, 29.70, 29.74, 29.91, 30.25, 30.74, 31.94, 31.97, 34.40 (C-2–C-8, C-11–C-17 & C-2”–C-15”), 50.19 (C-7’), 59.08 (C-31’), 64.22 (C-1”), 64.47 (C-1’), 69.56 (C-8’), 70.08, 70.52, 70.55, 70.60, 70.67 (C-9’–C-30’), 80.75 (C-9 & C-10), 123.85 (C-3’), 145.56 (C-2’), 174.03 (C-1).

#### 2.8.9. Synthesis of Compounds **22**–**23**

A solution of lactose azide (**17**) (2.2 equiv.) in *t*-butanol:water (2:1, 30 mL) was prepared, to which compound **6**/**7** (0.1 g, 1 equiv.) was added and the reaction mixture was stirred at 50 °C. After 10 min, copper powder (4 equiv.) was added and the mixture was allowed to stir for 48 h. Progress of reaction was monitored using TLC. Upon completion, the reaction mixture was cooled, diluted with ethanol (30 mL), and filtered through celite. The solvent was evaporated and purified using dialysis bag of 500 and 1000 Da to get pure compound (**22**/**23**) in 75–78% isolated yield.

Compound **22**: The click reaction of compound **7** with **17** gave compound **22** as a white solid in 75% yield by following the general procedure.

**IR** (**KBr**) **ν_max_**: 3340, 3050, 2914, 2839, 1745, 1417, 1262, 1144, 848, 730, 700 cm^−1^; **^1^H NMR** (**400 MHz**,**DMSO-*d_6_***): δ 0.81 (t, 3H, *^3^J* = 7.2 Hz, H-18), 1.21–1.47 (m, 26H, H-3–H-8, H-11–H-17), 2.25 (t, 2H, *^3^J* = 7.2 Hz, H-2), 2.56–2.67 (m, 2H, H-2”), 3.42–3.59 (m, 22H, H-5’–H-8’, H-11’–H-15’, H-9 & H-10), 3.71 (m, 4H, H-9’), 4.23 (d, 2H, *^3^J* = 6.8 Hz, H-10’), 4.28 (t, 2H, *^3^J* = 5.9 Hz, H-1”), 4.48–4.63 (m, 6H, 12 × OH), 4.94 (s, 4H, H-1’), 5.1 (s, 2H, 2 × OH), 5.57 (d, 2H, *^3^J* = 9.2 Hz, H-4’), 8.22 (s, 2H, H-3’); **^13^C NMR** (**100.5 MHz**, **DMSO-*d_6_***): δ 14.18 (C-18), 22.63, 24.81, 28.92, 29.16, 29.26, 29.49, 29.63, 29.79, 31.81, 33.86 (C-2–C-8, C-11–C-17 & C-2”), 56.42 (C-1’’), 60.58 (C-9’), 60.88 (C-15’), 68.61 (C-1’), 70.28, 71.06, 72.25, 73.76, 74.48, 75.71, 76.12, 78.26 (C-5’–C-8’ & C-11’–C-14’), 80.29 (C-9 & C-10), 87.45 (C-4’), 102.59, 103.43, 104.33 (C-10’), 123.63 (C-3’), 144.94 (C-2’), 173.18 (C-1).

Compound **23**: The click reaction of compound **6** with **17** gave compound **23** as a white solid in 78% yield by following the general procedure.

**IR** (**KBr**) **ν_max_**: 3374, 3055, 2929, 2849, 1726, 1427, 1241, 895, 730, 703; **^1^H NMR** (**400 MHz**, **DMSO-*d_6_***): δ 0.85 (m, 6H, H-18 & H-16”), 1.23–1.54 (m, 54H, H-3–H-8, H-11–H-17 & H-2”–H-15”), 2.27 (t, 2H, *^3^J* = 7.2 Hz, H-2), 3.48–3.89 (m, 26 H, H-5’–H-9’, H-11’–H-15’, H-9 & H-10), 3.99 (d, 2H, *^3^J* = 6.5 Hz H-1’’), 4.26 (d, 2H, *^3^J* = 6.9 Hz, H-10’), 4.53–4.56 (m, 4H, H-1’), 4.64–4.68 (m, 6H, 6 × OH), 4.83 (d, 2H, *^3^J* = 4.6 Hz, 2 × OH), 4.89 (s, 2H, 2 × OH), 5.14 (d, 2H, *^3^J* = 4.1 Hz, 2 × OH), 5.55 (d, 2H, *^3^J* = 5.9 Hz, 2 × OH), 5.63 (d, 2H, *^3^J* = 9.3 Hz, H-4’), 8.26 (s, 2H, H-3’); **^13^C NMR** (**100.5 MHz**, **DMSO-*d_6_***): δ 14.41 (C-18 & C-16”), 22.56, 24.99, 25.81, 28.57, 28.96, 29.03, 29.16, 29.36, 29.47, 29.75, 30.02, 31.76, 34.04 (C-2–C-8, C-11–C-17 & C-2”–C-15”), 60.64 (C-9’), 60.86 (C-15’), 63.77 (C-1”), 64.07 (C-1’), 68.60, 71.03, 72.21, 73.72, 75.67, 76.05, 78.20, 80.17 (C-5’–C-8’ & C-11’–C-14’), 80.32 (C-9 & C-10), 87.41 (C-4’), 104.28 (C-10’), 123.44 (C-3’), 144.90 (C-2’), 173.41 (C-1).

## 3. Results and Discussion

A systematic synthetic methodology was adopted to produce nonionic amphiphiles. The process involved esterification of oleic acid to produce ethyl (*Z*)-octadec-9-enoate (**2**). A peak at around 1720 cm^−1^ in the IR spectrum corroborates the esterification of oleic acid. The olefinic bond of the resulting compound **2** was subjected to epoxidation using *m*CPBA, followed by ring opening under acidic conditions, resulting in the formation of diol (**4**) (Scheme 1a). The two hydroxyl groups in compound **4** were propargylated using propargyl bromide to obtain compound **5**. During the reaction, the ester group was also hydrolyzed due to the strongly basic conditions generated by NaH. The appearance of peaks at δ 4.25 and δ 2.41 ppm in the ^1^H NMR spectrum of compound **5** corresponding to the methylene and alkyne protons, respectively, confirms its formation (Figure S4). Compound **5** was coupled with appropriate alkyl/fluoroalkyl chains via an ester linkage using EDC.HCl and DMAP as coupling reagents, by following an earlier published procedure from our group, ^17^ yielding the hydrophobic precursors **6**/**7**. The observance of peak at around 1725 cm^−1^ in the IR spectrum supports the ester coupling in compounds **6** and **7**. In the ^1^H NMR spectrum of compound **6**, a triplet for methyl protons at δ 0.88 ppm, along with the peaks for other methylene protons at δ 1.23–1.45 ppm in the proton NMR spectrum confirms the presence of hydrophobic alkyl chain (Figure S5). Similarly, the ^19^F NMR spectrum of compound **7** establishes the presence of a fluoroalkyl chain. Monomethoxy polyethylene glycol (mPEG) (**8**/**9**) was used as the hydrophilic head group. mPEG-azides (**12**/**13**) were first synthesized through mesylation followed by azidation of commercially available alcohol of mPEG-550 (**8**) and mPEG-750 (**9**), following a previously established procedure by our group (Scheme 1b) [28]. The presence of azide group in compound **12** and **13** was confirmed by the appearance of peak at around 2150 cm^−1^ in the IR spectrum.

**Scheme 1:**
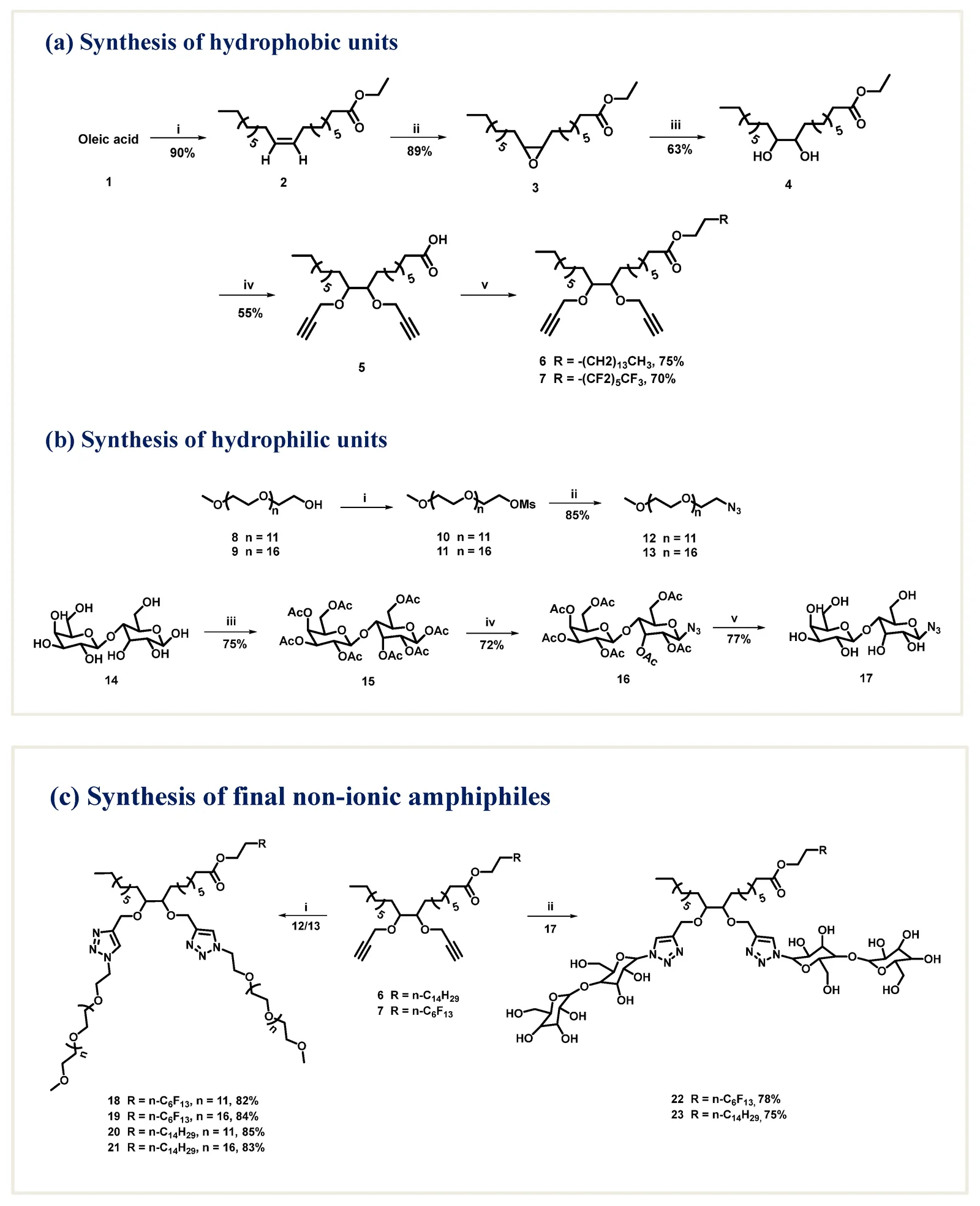
(**a**) Synthesis of hydrophobic units: (i) Conc. H_2_SO_4_, ethanol, 80 °C, 24 h; (ii) m-CPBA, DCM, rt, 2 h; (iii) Conc. H_2_SO_4_, THF:H_2_O (1:1), rt, 12 h; (iv) NaH, propargyl bromide, DMF, rt, 24 h; (v) n-alkyl/perfluoroalkyl alcohol, EDC.HCl, DMAP, DCM, rt, 24 h. (**b**) Synthesis of hydrophilic units: (i) mesyl chloride, triethyl amine, DCM, 0-25 °C, 3 h; (ii) sodium azide, DMF, 80 °C, 20 h; (iii) Ac_2_O, NaOAc, reflux, 2 h; (iv) TMSN_3_, SnCl_4_, DCM, 25 °C, 12 h; (v) NaOMe, MeOH, 25 °C, 12 h. (**c**) Synthesis of final non-ionic amphiphiles: (i) [Cu(PPh_3_)_3_]Br, DIPEA, DMF, 70 °C, 24 h; (ii) Cu powder, tert-butanol, H_2_O, 50 °C, 48 h.

For incorporating carbohydrate moiety, lactose azide (**17**) was prepared from D-lactose (**14**) by first converting it into lactose octaacetate (**15**). Subsequent treatment of compound **15** with SnCl_4_/TMSN_3_ furnished hepta-acetoxy lactose azide (**16**). In the IR spectrum of **16**, the disappearance of the broad O-H stretching band at 3319 cm^−1^ and the appearance of a sharp azide band at 2100 cm^−1^ confirmed its formation. Finally, deacetylation of **16** with sodium methoxide yielded lactose azide (**17**) as the target building block (Scheme 1b).

Compounds **6** and **7** were then click coupled with the mPEG-550/750 azide (**12**/**13**) and lactose azide (**17**) by using click chemistry to obtain six different oleic-acid-based nonionic amphiphiles (**18**–**23**) as shown in Scheme 1c. The structures of the six targeted amphiphiles and their precursors were thoroughly confirmed by using a combination of ^1^H, ^13^C, and ^19^F, HRMS, and IR data. The PEG protons were identified in the region of δ 3.50–3.60, while the protons of lactose moiety were observed in the range δ 3.40–5.60 ppm, with their corresponding carbon signals at δ 65–72 and δ 60–80 ppm, respectively, supporting the successful click coupling with the mPEG and lactose azides (Figures S7–S12). Additionally, the disappearance of azide peak at 2100 cm^−1^ provided further confirmation of the click reaction for the resulting amphiphiles.

### 3.1. Physicochemical Characterization

The synthesized amphiphiles were found to be soluble in aqueous medium. DLS measurements were used to examine their self-organization behavior, and TEM analysis verified the morphology of the self-assembled supramolecular frameworks. A fluorescence approach was used to measure the critical aggregation concentration [29].

#### 3.1.1. Critical Aggregation Concentration Measurement

##### Determination of CAC by Fluorescence Sprectroscopy

Critical Aggregation Concentration (CAC) is a characteristic of amphiphilic compounds. The CAC for amphiphiles **18**–**23** was evaluated using Nile red as a fluorescence probe. The fluorescence intensity of the fluorescent dye varies in response to changes in the hydrophobic microenvironment, exhibiting the solvatochromic phenomenon. At a specific concentration, the amphiphiles begin to self-aggregate, forming a hydrophobic cavity capable of encapsulating the dye. This led to a significant rise in the fluorescence intensity of Nile red. The plot depicting the logarithm of the amphiphile concentration versus the fluorescence intensity of the encapsulated Nile red was generated (Figure 1), and the point at which the curve shifts direction denotes the CAC value of the amphiphile. The CAC values of fluorinated amphiphiles **18**, **19**, and **22** were observed to be 0.12 mg/mL, 0.15 mg/mL, and 0.13 mg/mL, respectively, which are lower than those of their non-fluorinated analogs, **20**, **21**, and **23**, with CAC values of 0.22 mg/mL, 0.32 mg/mL, and 0.14 mg/mL (Table 1). This difference is attributed to the enhanced hydrophobicity and micellar stability of fluorinated amphiphiles. This may be attributed to orderly packing of the rigid fluoroalkyl chains in their all-trans helical conformation [21].

##### Determination of CAC Using Count Rate Method by DLS

To further validate the CAC values obtained using the Nile red fluorescence method, the CAC of amphiphile **21** was determined independently using the DLS count-rate method (Figure S20) [30]. For this analysis, the count rate (kcps) was plotted as a function of the logarithm of amphiphile concentration, and the CAC was determined from the intersection of the two linear regions corresponding to the pre-aggregation and post-aggregation regimes. The CAC obtained by using the DLS count-rate method was 1.45 × 10^−4^ M, which closely matches the value of 1.48 × 10^−4^ M obtained by using the fluorescence method. The two measurements differed by only about 2%, indicating excellent agreement between the two independent approaches. This close correlation further supports the reliability of the CAC values obtained by the Nile red fluorescence method.

#### 3.1.2. DLS Study

The supramolecular self-organization behavior of the synthesized amphiphiles in aqueous solution was investigated using DLS measurements at a concentration of 5 mg/mL [31]. The DLS size distribution graph for amphiphiles **18**–**23** is depicted in Figure 2. The size distribution profile for nanocarriers exhibited a bimodal pattern in intensity (Figure S13) and a monomodal pattern in volume and number. The two peaks observed in the intensity distribution profile are attributed to micelles and micellar aggregates. Meanwhile, the single peak in volume and number (Figure 2), which corresponds to the smaller peak in intensity and is associated with micelles, suggests that micelles are the dominant species present in the aqueous solution. The data obtained from DLS measurements, summarized in Table 1, reveal that the particle size of the self-assembled nanosized species formed by aggregation in aqueous solution is approximately 6–12 nm [32].

#### 3.1.3. TEM Study

Transmission electron microscopy (TEM) was employed to examine the self-assembled nanostructures formed by amphiphiles containing an identical hydrophobic moiety but different hydrophilic units (Figure 3). The mPEG-based amphiphile exclusively formed worm-like micelles with characteristic dimensions of approximately ~10 nm (Figure 3a), consistent with its high chain flexibility and strong hydration, which favor one-dimensional micellar growth. In contrast, the lactose-based amphiphile predominantly formed spherical micelles with sizes also centered around ~10 nm (Figure 3c), as the rigid carbohydrate head groups limit micellar rearrangement and promote compact assemblies [33]. The sizes observed by TEM are in good agreement with the hydrodynamic diameters obtained from DLS, confirming the formation of well-defined nanoscale assemblies in aqueous media.

#### 3.1.4. Guest Encapsulation Behavior

Amphiphilic molecules exhibit a remarkable ability to self-assemble into structured aggregates in aqueous environments, creating hydrophobic cavities that can effectively encapsulate guest molecules, thereby enhancing their solubility and targetability [34]. This encapsulation behavior of the amphiphiles was primarily demonstrated using the dye Nile red, employing the thin film method. Nile red is extensively used as a guest molecule to evaluate the transport capabilities of nanocarriers, owing to its pronounced absorbance in lipophilic surroundings. This fluorescent dye, being neutral, hydrophobic, and solvatochromic, is recognized for its tendency to form *H*- and *J*- type aggregates beyond a certain concentration due to the possibility of self-aggregation. Thus, as per the earlier studies from our group, 0.12 mg Nile red was used for encapsulation in a solution comprising 5 mg/mL of amphiphile [35].

Additionally, the hydrophobic drugs nimodipine and curcumin were also used to evaluate the encapsulation potential of the amphiphiles **18**–**23**. Nimodipine is a dihydropyridine calcium channel blocker developed by Bayer AG in 1983 and is known for its efficacy in the central nervous system, particularly due to its ability to cross the blood–brain barrier, making it valuable for treating conditions related to cerebral blood flow [36]. However, its hydrophobic nature limits its solubility in water (0.4 mg/L), and the commercial formulations require a cosolvent system comprising polyethylene glycol (62.5 g) and ethanol (37.5 g) to solubilize 50 mg of Nimodipine [37]. Curcumin, a natural polyphenolic compound derived from *Curcuma longa*, is widely utilized for its anti-inflammatory, antioxidant, anticancer, antimicrobial, and neuroprotective properties, and is also used as a natural food colorant and nutraceutical ingredient [38,39]. Despite these advantages, curcumin too suffers from extremely low aqueous solubility, rapid degradation in aqueous environments, and very poor oral bioavailability (<1%) [40,41]. Furthermore, curcumin undergoes extensive sulfation and glucuronidation, especially in the liver, leading to poor absorption, rapid metabolism, and fast excretion, which collectively account for its low levels in blood and tissues [42]. Therefore, the nanocarrier- mediated encapsulation strategy is used to enhance their solubility besides facilitating its encapsulation and circulation in biological systems, while enabling efficient loading and nanoscale dispersion in aqueous media (Figure 4). We have successfully encapsulated nimodipine and curcumin by taking 1.6 mg and 0.5 mg of these, respectively, in 5 mg/mL aqueous solutions of the synthesized amphiphiles **18**–**23** [43]. UV-Vis spectroscopy confirmed the negligible intrinsic aqueous solubility of curcumin and nimodipine, with absorbance increasing ~11.8-fold and ~47.5-fold, respectively, upon amphiphile-mediated solubilization (Figure S21).

**Table 2:** Loading data of nanocarriers (**18**–**23**).

Amphiphiles	Transport Efficiency (mg/g)			Transport Capacity (mmol/mol)			Encapsulation Efficiency (%)		
	Nile Red	Curcumin	Nimodipine	Nile Red	Curcumin	Nimodipine	Nile Red	Curcumin	Nimodipine
**18**	1.01	17.11	18.59	6.04	88.51	84.69	4.19	17.11	5.81
**19**	0.92	10.25	11.65	6.66	64.15	64.21	3.81	10.25	3.64
**20**	1.28	15.99	19.17	7.13	76.71	80.95	5.35	15.99	6.00
**21**	1.22	18.71	11.04	8.32	110.06	57.19	5.09	18.71	3.45
**22**	0.77	6.59	8.42	3.51	25.96	29.21	3.23	6.59	2.63
**23**	1.44	11.92	16.85	5.93	42.44	76.75	6.00	11.92	5.26

The encapsulation potential of Nile red, nimodipine, and curcumin is influenced by the hydrophobic chain and hydrophilic head groups, which dictate the hydrophilic–lipophilic balance (HLB) of the amphiphiles. HLB values of the amphiphiles synthesized fall in the range 9.8 to 14 (Table 1), affecting drug/dye loading behavior. Lactose-based fluoroalkyl amphiphiles (**22** & **23**) exhibited the lowest HLB values and poor encapsulation. In contrast, alkylated lactose amphiphile showed better results, perhaps due to enhanced flexibility in alkyl chains. For nimodipine, amphiphiles with mPEG-550 (**18** & **20**) provided superior encapsulation, with optimal HLB around 12 for fluorinated types. Curcumin encapsulation also favored alkylated structures with mPEG-750 (**19** & **21**) (Table 2). Overall, flexible alkylated amphiphiles performed better across a broader HLB range, while fluorinated options were sensitive to HLB variations. The study emphasizes the need for carefully designed amphiphiles to optimize loading of diverse hydrophobic therapeutics.

#### 3.1.5. Cytotoxicity Study

The cytotoxicity of the synthesized amphiphiles was assessed using the CCK-8 assay on L929 fibroblast cells over a concentration range between 0.50 and 0.015625 mg mL^−1^ (Figure 5). All amphiphiles, except **18**, **19**, **21** at the highest concentration, exhibited negligible cytotoxicity, with cell viability remaining above the acceptable threshold of >80% across the tested concentrations. Notably, no concentration-dependent reduction in cell viability was observed up to 0.25 mg/mL^−1^, confirming the inherent biocompatibility of both alkylated and fluorinated systems. A significant improvement in cell viability was observed for fluorinated amphiphiles upon replacement of PEG with a carbohydrate-based hydrophilic segment (lactose). While PEG-containing fluorinated amphiphile (**18** & **19**) showed approximately 50% viability at 0.50 mg L^−1^, lactose-substituted analogue (**22**) exhibited higher viability (~80%) at the same concentration. This enhancement is attributed to the superior biocompatibility of carbohydrate-based hydrophilic segments, which improve surface hydration and reduce unfavorable interactions with cellular membranes. Overall, these results demonstrate that the synthesized amphiphiles are biocompatible, supporting their potential as safe nanocarriers for drug delivery applications.

#### 3.1.6. Enzymatic Release Study

The use of enzyme-triggered release mechanisms in drug delivery systems represents a significant advancement in precision medicine, offering targeted and controlled release of therapeutic agents. In this study, we synthesized amphiphiles with ester functionalities that are susceptible to enzymatic cleavage by hydrolase. We utilized this property by performing ester hydrolysis of the amphiphiles using an immobilized enzyme, *Candida antarctica* lipase B (CAL B), commercially available as Novozym 435 [44]. We investigated the rupture of the amphiphilic micellar architecture by examining the time-dependent release of an encapsulated dye, Nile red. In the experiment, a solution of Nile red-encapsulated amphiphile was treated with 200 wt% Novozym 435, and a few drops of n-butanol were added to capture the acid released from the hydrolysis of the amphiphile esters. The release studies were conducted by incubating the sample under dark conditions at 37 °C and monitoring the fluorescence intensity of the dye over time. As the amphiphilic architecture ruptured, the dye moved from a lipophilic environment, resulting in a gradual decrease in fluorescence intensity. Complete release of the dye was observed within 66 h in the amphiphilic system **21**, as illustrated in Figure 6a. A control experiment without the enzyme showed no noticeable change in the fluorescence intensity, indicating that a negligible amount of Nile red was released (Figure 6b).

## 4. Conclusions

In this study, we have successfully designed and synthesized six novel nonionic amphiphiles (**18**–**23**) by incorporating mPEG/lactose as the hydrophilic head group alongside either alkyl or fluoroalkyl chains as lipophilic segments. The structural identity of all synthesized amphiphiles was thoroughly confirmed through comprehensive spectroscopic characterization, including ^1^H, ^13^C, and ^19^F NMR analysis. A comparative evaluation of the supramolecular self-assembly behavior revealed that fluorocarbon-tailed amphiphiles exhibited significantly lower critical aggregation concentrations (CAC) relative to their alkyl-chain counterparts. Dynamic light scattering (DLS) and transmission electron microscopy (TEM) analysis confirmed that all amphiphiles self-assemble into well-defined nanoscale micellar structures. Cytotoxicity assessments L929 fibroblast cells demonstrated that none of the amphiphiles exhibited measurable toxicity at concentrations up to 0.5 mg/mL, affirming their biocompatibility. Among the synthesized systems, alkylated amphiphiles displayed superior encapsulation efficiency for hydrophobic dyes and drugs, attributed to the inherent flexibility of their hydrophobic cores, which facilitates favorable guest–host interactions. Enzyme-triggered release experiments revealed complete cargo release, reflected by a 100% decline in the fluorescence intensity within 66 h. Collectively, these findings demonstrate the robust encapsulation capacity and stimuli-responsive release behavior of these amphiphilic nanoarchitectures, establishing them as promising platforms for the development of advanced nanocarriers in biomedical applications.

## Figures and Tables

**Figure 1:**
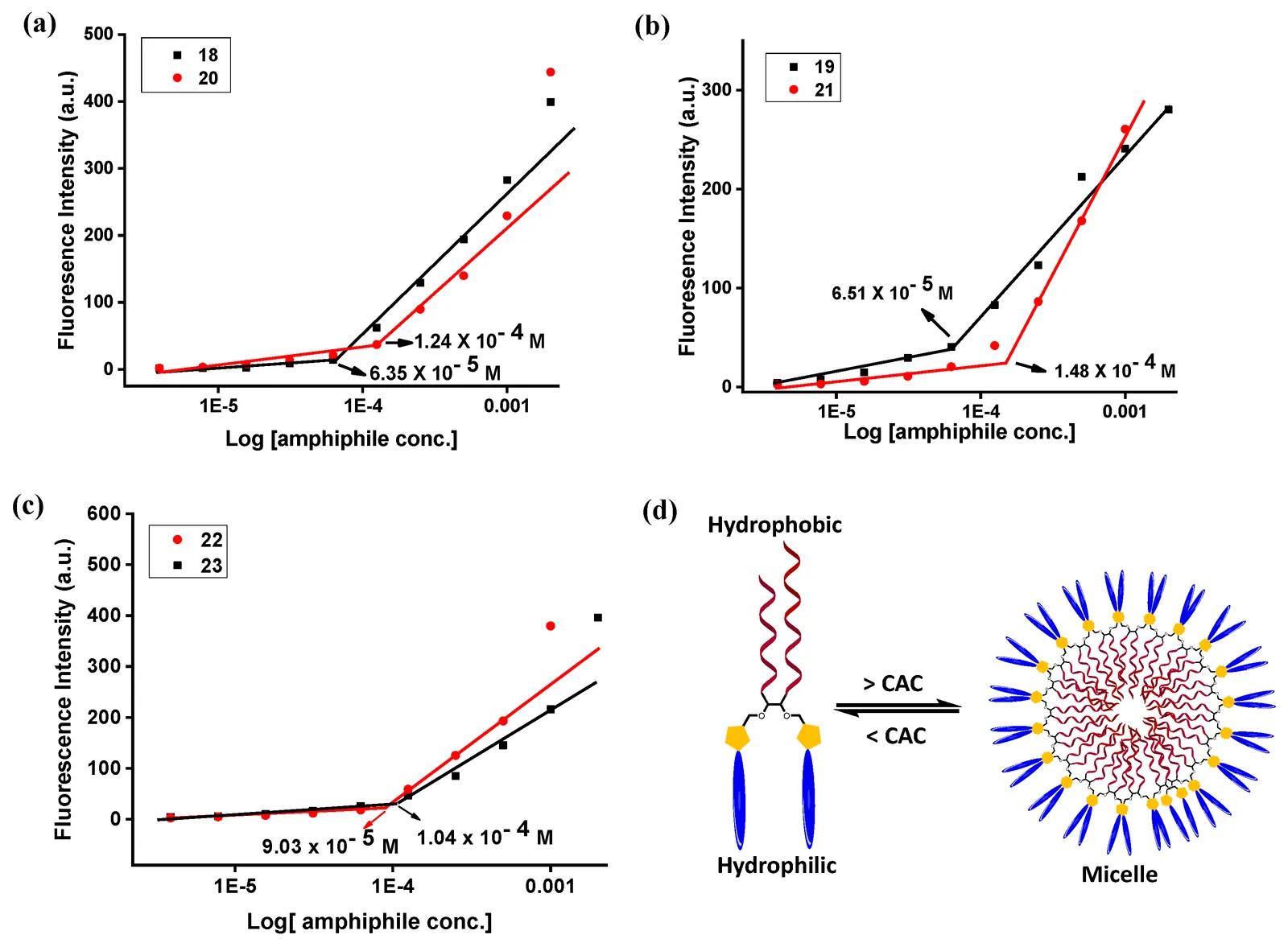
Fluorescence intensity as a function of log[amphiphile concentration] for (**a**) **18**, **20**; (**b**) **19**, **21**; (**c**) **22**, **23** in water at 25 °C and (**d**) schematic representation of amphiphile above and below CAC.

**Figure 2:**
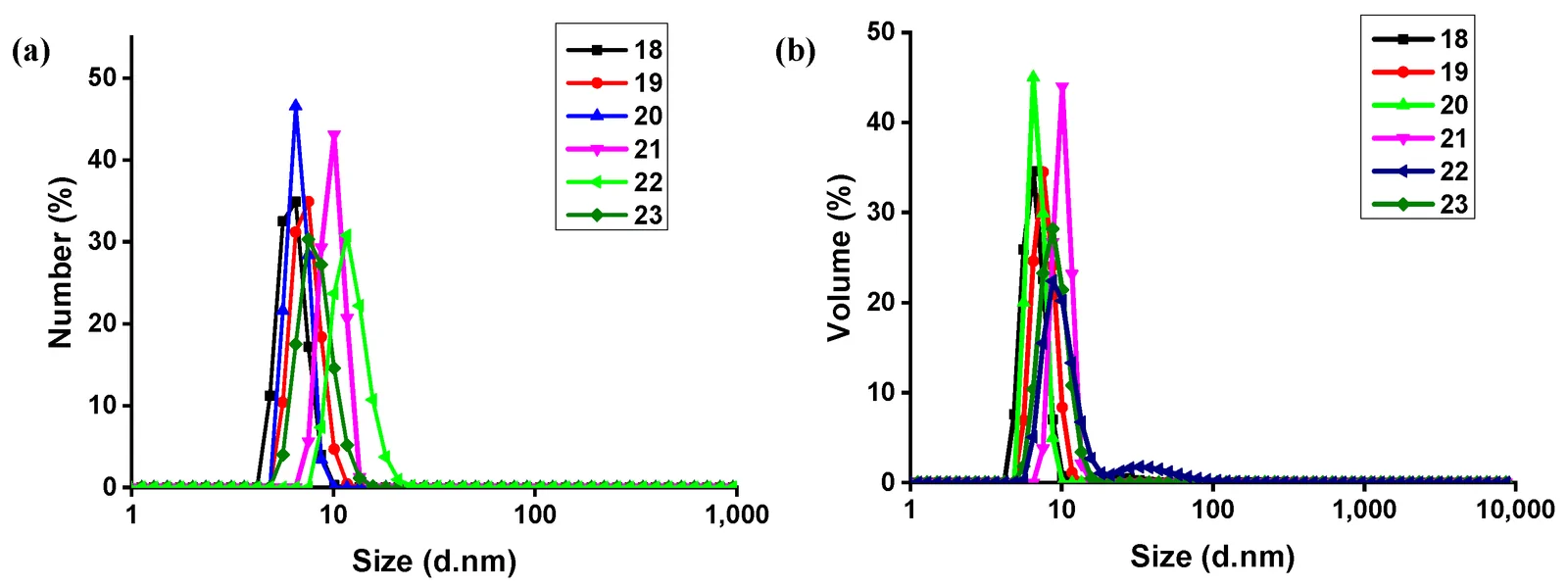
Dynamic light scattering (DLS) profiles of amphiphiles **18**–**23** in aqueous medium (**a**) number and (**b**) volume distributions.

**Figure 3:**
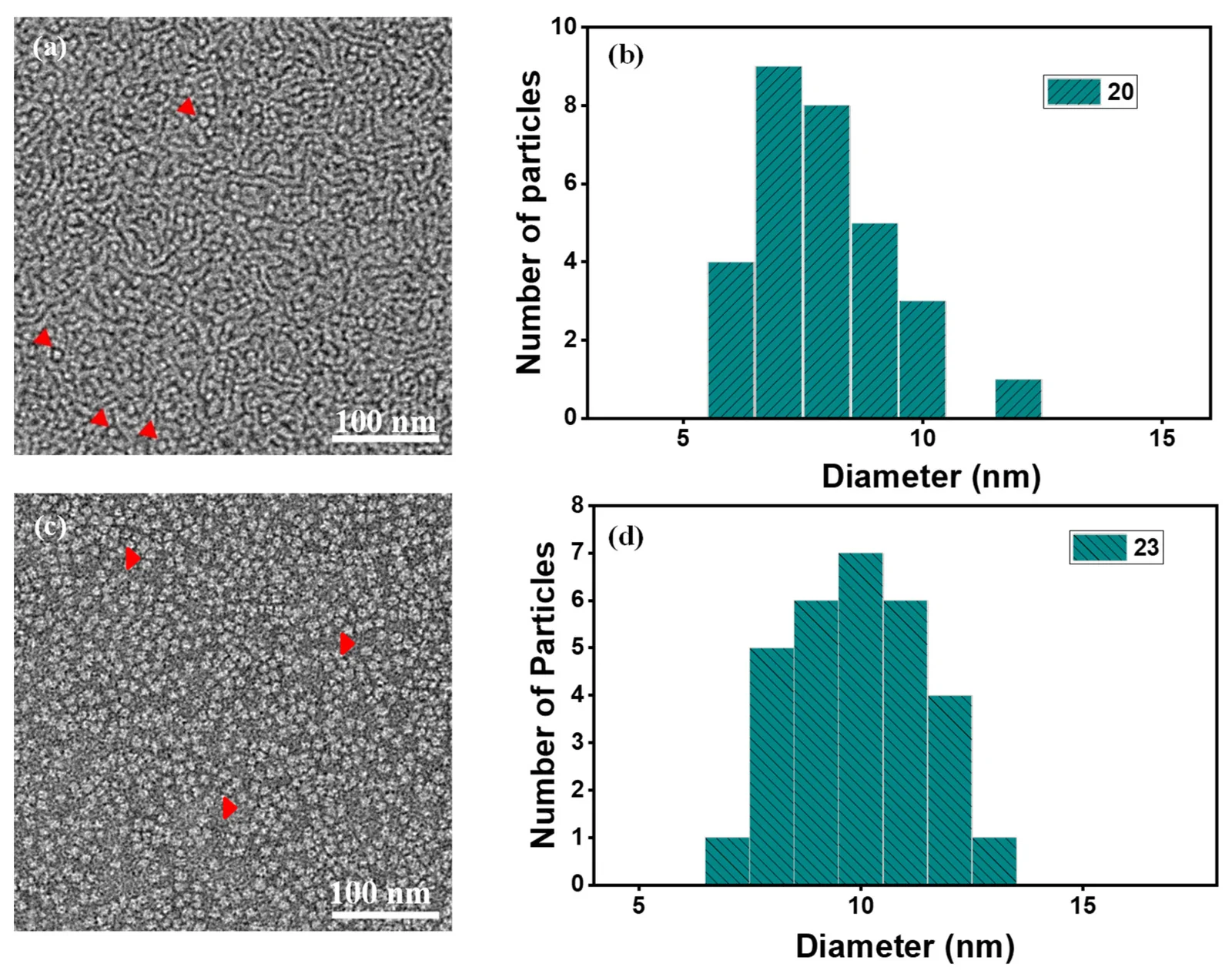
(**a**) TEM image of amphiphile **20** displaying worm-like micellar aggregates; (**b**) corresponding micellar size-distribution histogram, indicating an average size of ~10 nm. (**c**) TEM image of amphiphile **23** showing spherical micelles; (**d**) corresponding size-distribution histogram derived from TEM measurements, also revealing an average diameter of ~10 nm.

**Figure 4:**
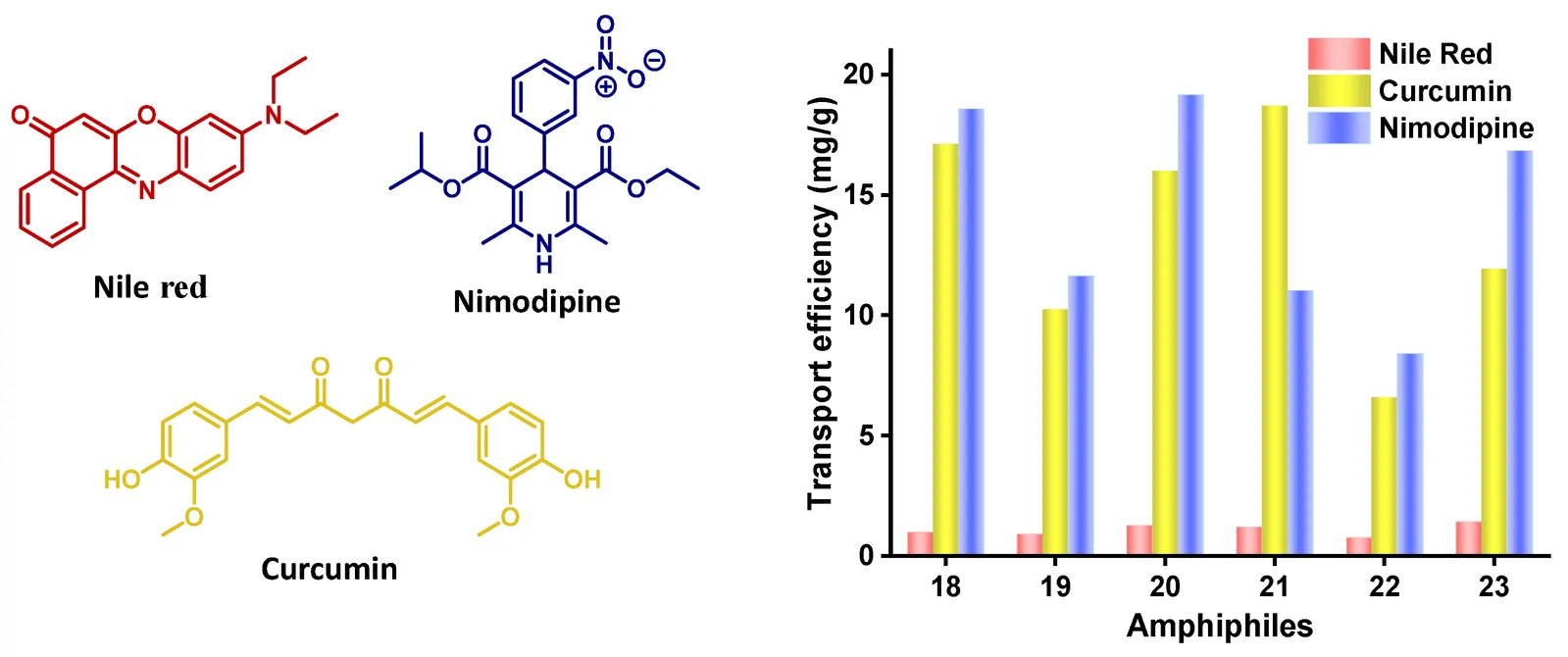
Structural representation of hydrophobic guests and transport efficiency of nanocarriers (**18**–**23**).

**Figure 5:**
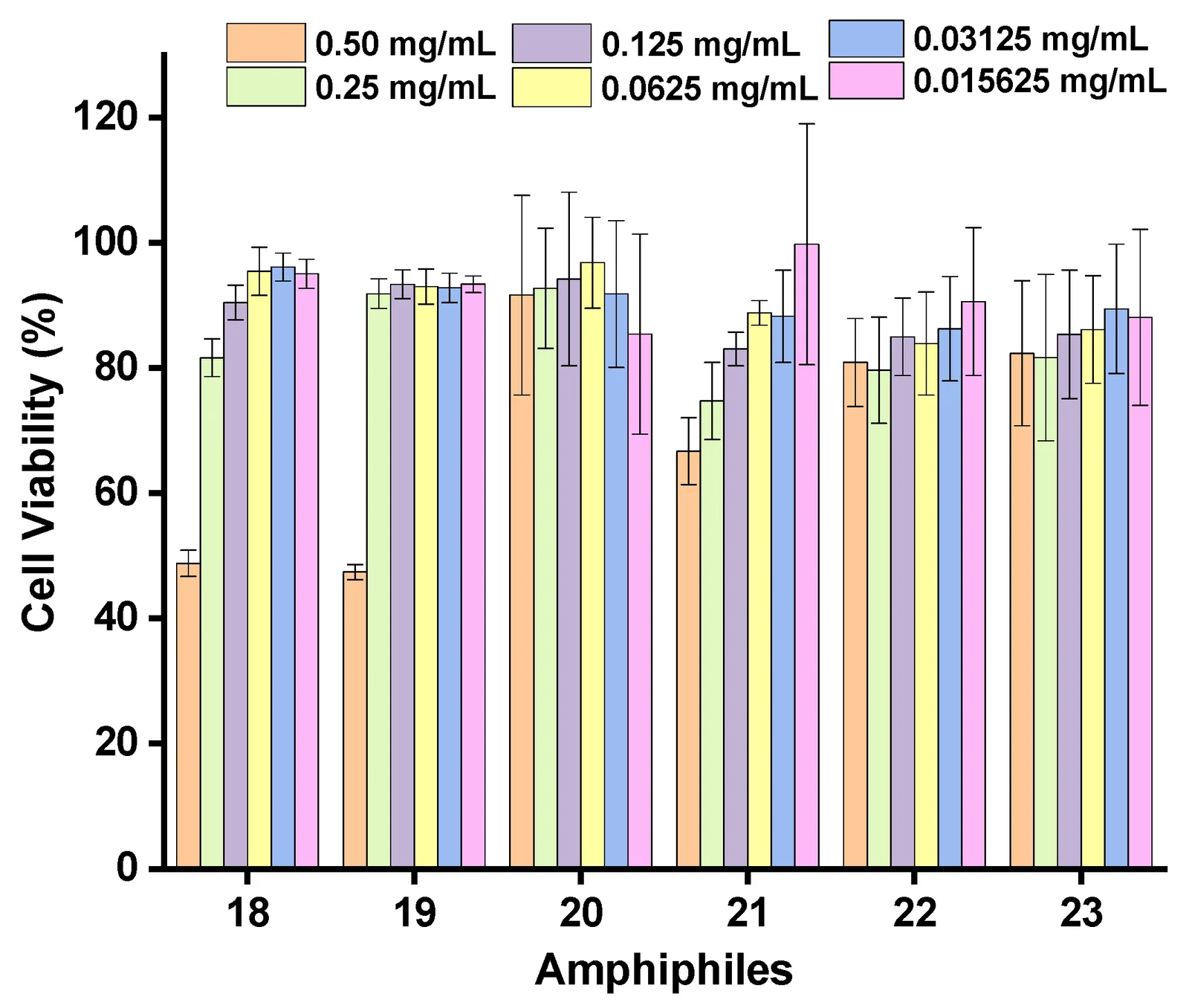
Effect of amphiphiles on cell viability of L929 cells by CCK-8 assay using a water-soluble tetrazolium salt.

**Figure 6:**
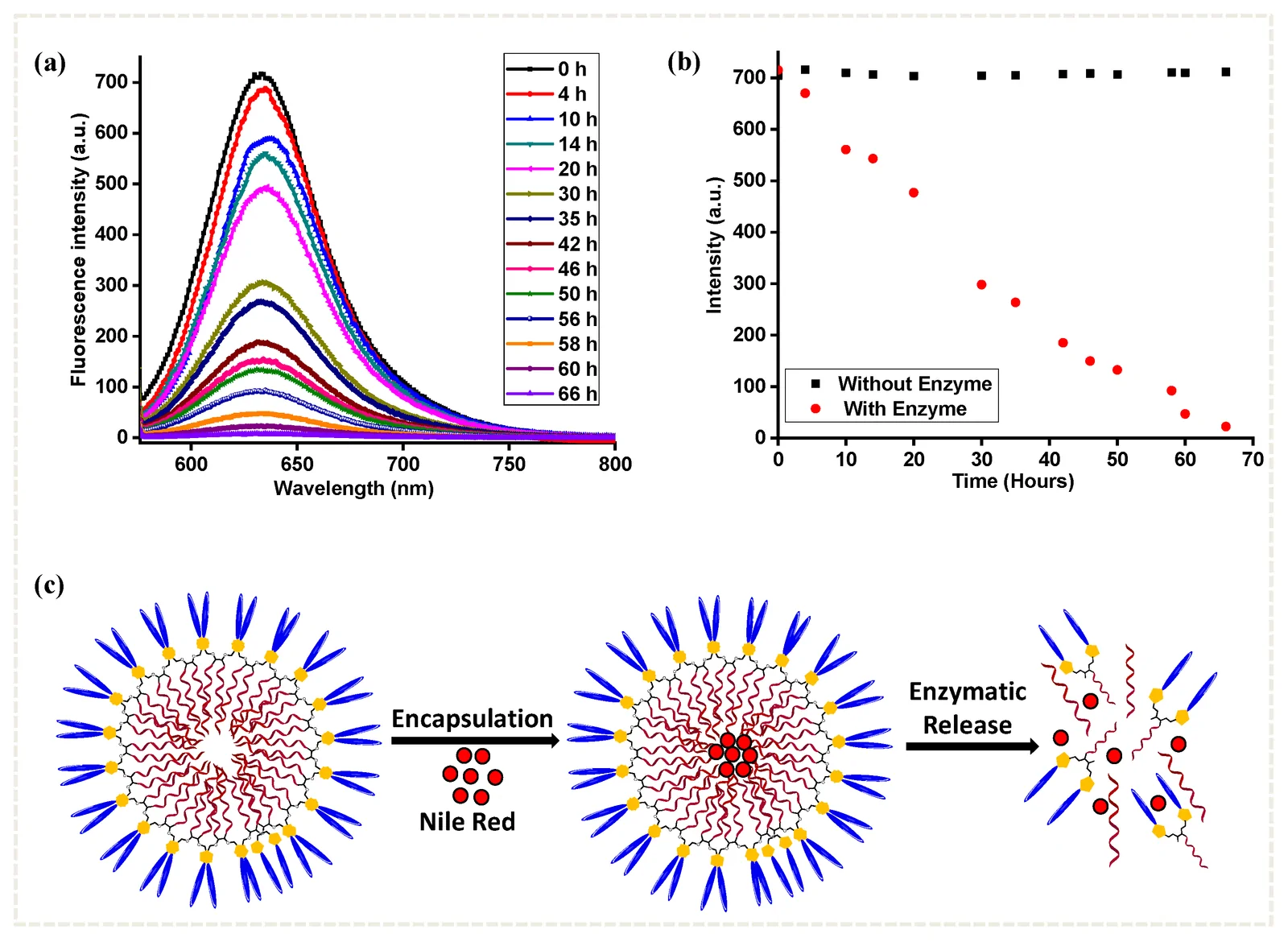
Release of Nile red encapsulated in nanocarrier **21** under physiological conditions. (**a**) Variation in fluorescence intensity with time of amphiphile-encapsulated Nile red in the presence of enzyme at 37 °C; (**b**) Comparison of intensity variation in the dye with time in the absence and presence of enzyme at 37 °C; (**c**) Graphical representation of the enzymatic release of dye from micelle.

**Table 1:** Aggregation behavior data of synthesized non-ionic amphiphiles (**18**–**23**).

Amphiphiles	^a^ HLB = 20 × M_h_/M_W_	CAC (mg/mL)	DLS (nm)	PDI
**C-F_8_**/**mPEG-550** (**18**)	12.18	0.12	6.5 ± 0.08	0.42
**C-F_8_**/**mPEG-750** (**19**)	13.38	0.15	7.3 ± 0.30	0.56
**C-16**/**mPEG-550** (**20**)	12.8	0.22	6.1 ± 0.12	0.57
**C-16**/**mPEG-750** (**21**)	14.1	0.32	10.2 ± 0.78	0.54
**C-F_8_**/**Lactose** (**22**)	9.85	0.13	12.1 ± 0.49	0.61
**C-16**/**Lactose** (**23**)	10.86	0.14	8.5 ± 0.11	0.49

^a^ Griffin equation; M_h_ = molecular weight of hydrophilic unit; M_w_ = molecular weight of amphiphile. DLS values are represented as mean ± standard deviation, n = 3 independent measurements.

## Data Availability

The original contributions in this study are included in the article/Supplementary Materials. Further inquiries can be directed to the corresponding authors.

## Supplementary Materials

The following supporting information can be downloaded at: https://www.mdpi.com/article/10.3390/pharmaceutics18101209/s1, Figure S1: ^1^H & ^13^C NMR spectra of compound **2** in CDCl_3_; Figure S2: ^1^H & ^13^C NMR spectra of compound **3** in CDCl_3_; Figure S3: ^1^H & ^13^C NMR spectra of compound **4** in CDCl_3_; Figure S4: ^1^H & ^13^C NMR spectra of compound **5** in CDCl_3_; Figure S5: ^1^H & ^13^CNMR spectra of compound **6** in CDCl_3_; Figure S6: ^1^H, ^13^C & ^19^F NMR spectra of compound **7** in CDCl_3_; Figure S7: ^1^H, ^13^C & ^19^F NMR spectra of compound **18** in CDCl_3_; Figure S8: ^1^H, ^13^C & ^19^F NMR spectra of compound **19** in CDCl_3_; Figure S9: ^1^H & ^13^C NMR spectra of compound **20** in CDCl_3_; Figure S10: ^1^H & ^13^C NMR spectra of compound **21** in CDCl_3_; Figure S11: ^1^H, ^13^C & ^19^F NMR spectra of compound **22** in DMSO-*d_6_*; Figure S12: ^1^H & ^13^C NMR spectra of compound **23** in DMSO-*d_6_*; Figure S13: DLS intensity distribution plots of amphiphiles (**18**–**23**); Figure S14: Quantification of Nile red encapsulation in amphiphiles (**18**–**23**) using UV absorption spectra; Figure S15: Quantification of nimodipine encapsulation in amphiphiles (**18**–**23**) using UV absorption spectra; Figure S16: Quantification of curcumin encapsulation in amphiphiles (**18**–**23**) using UV absorption spectra; Figure S17: Transport capacity of nanocarriers (**18**–**23**) for Nile red, nimodipine, and curcumin; Figure S18: Fluorescence emission spectra of amphiphile **22** at different concentrations; Figure S19: Fluorescence emission spectra of amphiphile **23** at different concentrations; Figure S20: Comparative determination of the critical aggregation concentration (CAC) of amphiphile **21** using two independent methods; Figure S21: UV–Vis absorption spectra of curcumin and nimodipine in aqueous medium in the absence and presence of the synthesized amphiphile. Figure S22: Time-dependent release of Nile red in the presence of enzyme.

## Abbreviations

The following abbreviations are used in this manuscript:

PEG	Poly Ethylene Glycol
DLS	Dynamic Light Scattering
TEM	Transmission Electron Microscope
CAC	Critical Aggregation Concentration
OA	Oleic Acid
UV-Vis	Ultraviolet Visible
mPEG	Monomethoxy polyethylene glycol
mCPBA	meta-chloroperoxybenzoic acid
NaH	Sodium hydride
NMR	Nuclear Magnetic Resonance
DMAP	4-Dimethylaminopyridine
EDC.HCl	1-Ethyl-3-(3-dimethylaminopropyl)carbodiimide hydrochloride
IR	Infrared
HLB	Hydrophilic Lipophilic Balance
CCK-8	CCK-8–Cell Counting Kit 8
CAL B	*Candida antarctica* lipase b
