# Supplementary material for: Oleic-Acid-Derived Nonionic Amphiphiles as Nanoscale Drug Delivery Vehicles
Source: Pharmaceutics. 2026 Sep 23;18(10):1209. doi: 10.3390/pharmaceutics18101209 (PMC13708122; doi:10.3390/pharmaceutics18101209)
Supplement: Supplementary file 1 [file pharmaceutics-18-01209-s001.zip › pharmaceutics-4529739-supplementary.pdf]

Supplementary data

Oleic Acid-Derived Nonionic Amphiphiles as Nanoscale Drug Delivery Vehicles

Sudhanshu Vats[a,b], Aarti[a,c], Natalie Hanheiser[c], Mathias Dimde[c,d], Abhishek K. Singh[c*], Sunil K. Sharma[a*]

[a]*Department of Chemistry, University of Delhi, Delhi-110 007, India*

[b] *Pt. Nekiram Sharma Government College Rohtak, Department of Higher Education, Haryana-124001, India*

[c]*Institut für Chemie und Biochemie, Freie Universität Berlin, Takustraße 3, 14195 Berlin, Germany*

[d]*Forschungszentrum für Elektronenmikroskopie, Core-Facility BioSupraMol, Institute of Chemistry and Biochemistry, Freie Universität Berlin, Fabeckstraße 36a, 14195 Berlin, Germany*

*Email: abhikmc@zedat.fu-berlin.de; sksharma@chemistry.du.ac.in

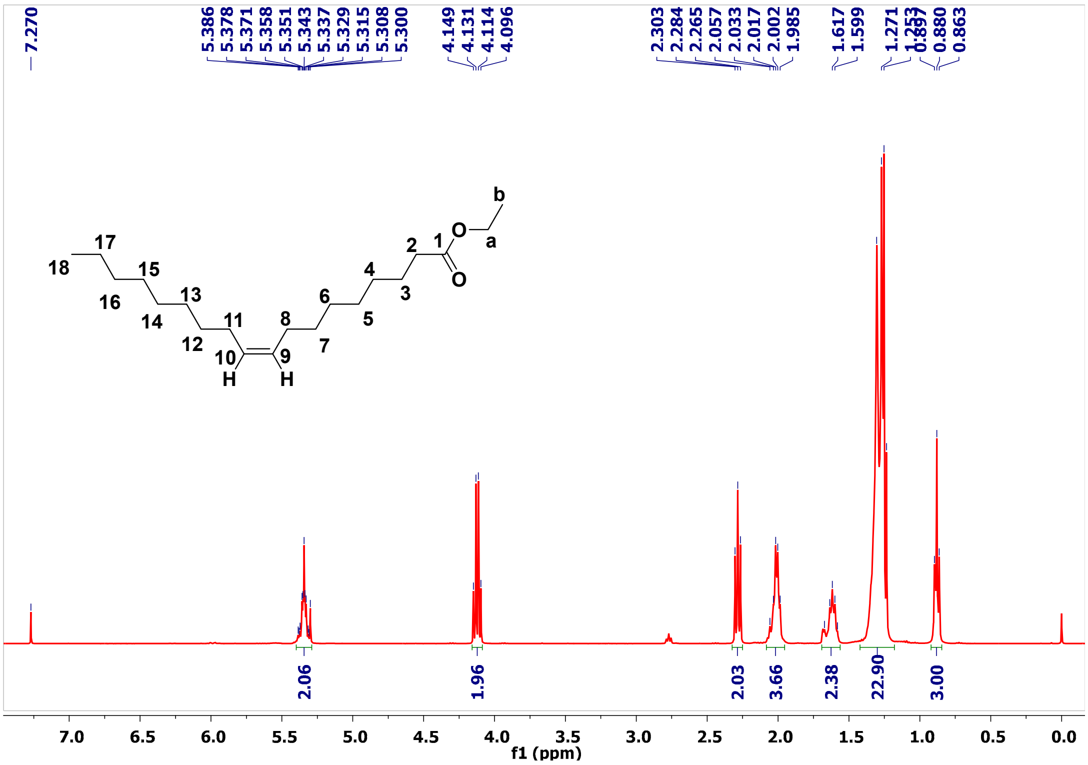

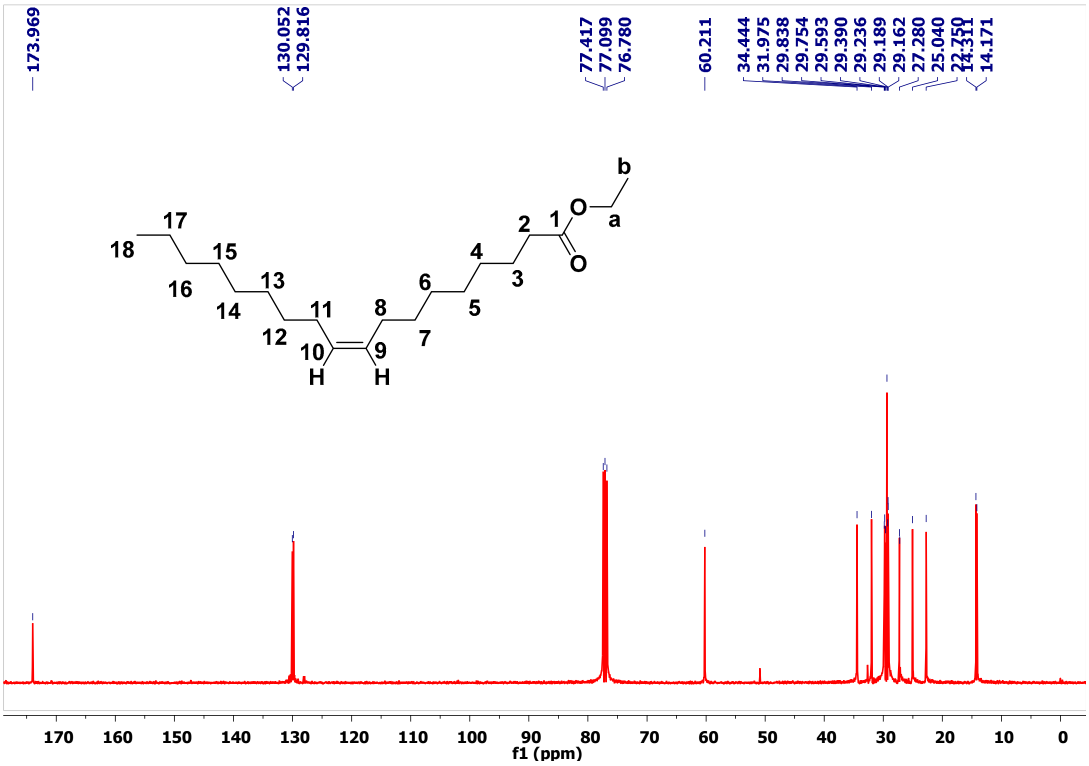

Figure S1. ^{1}H & ^{13}C NMR spectra of compound **2** in $CDCl_3$.

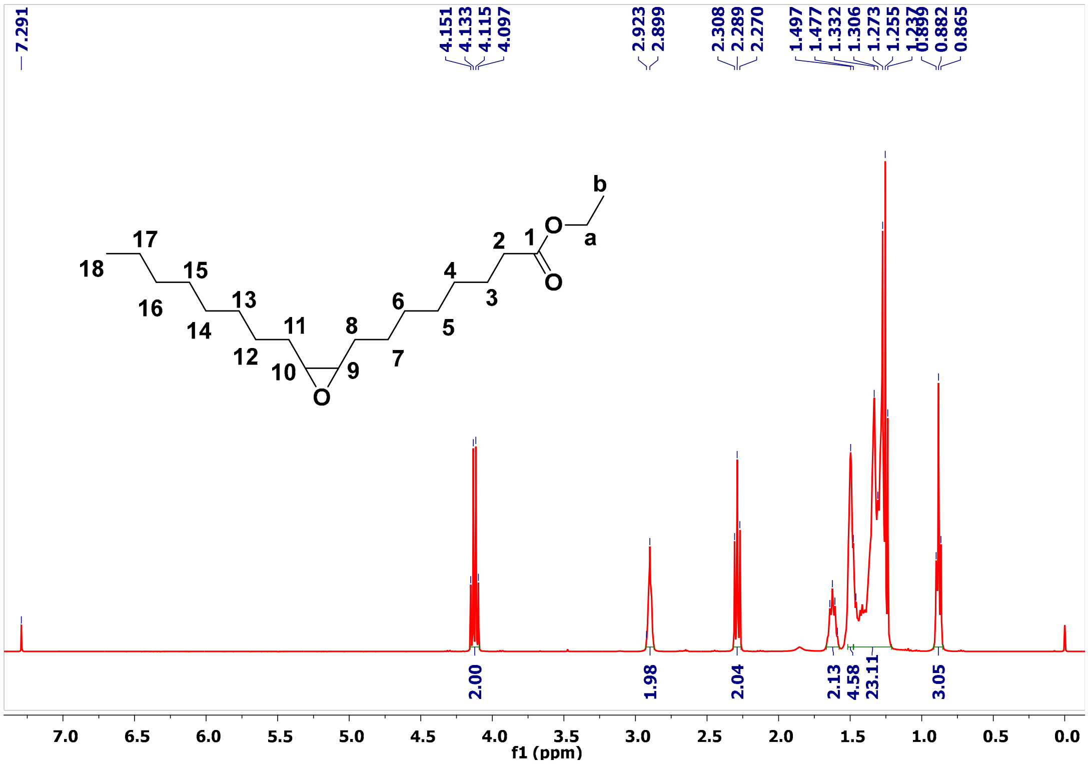

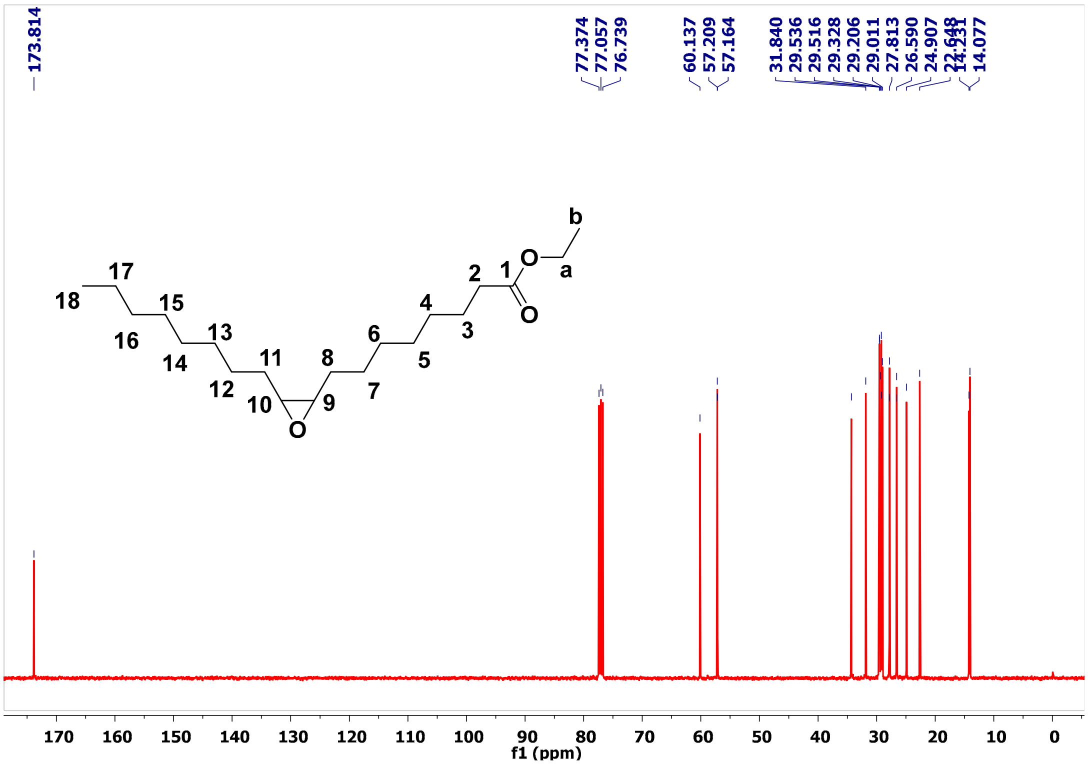

Figure S2. ^{1}H & ^{13}C NMR spectra of compound **3** in $CDCl_3$.

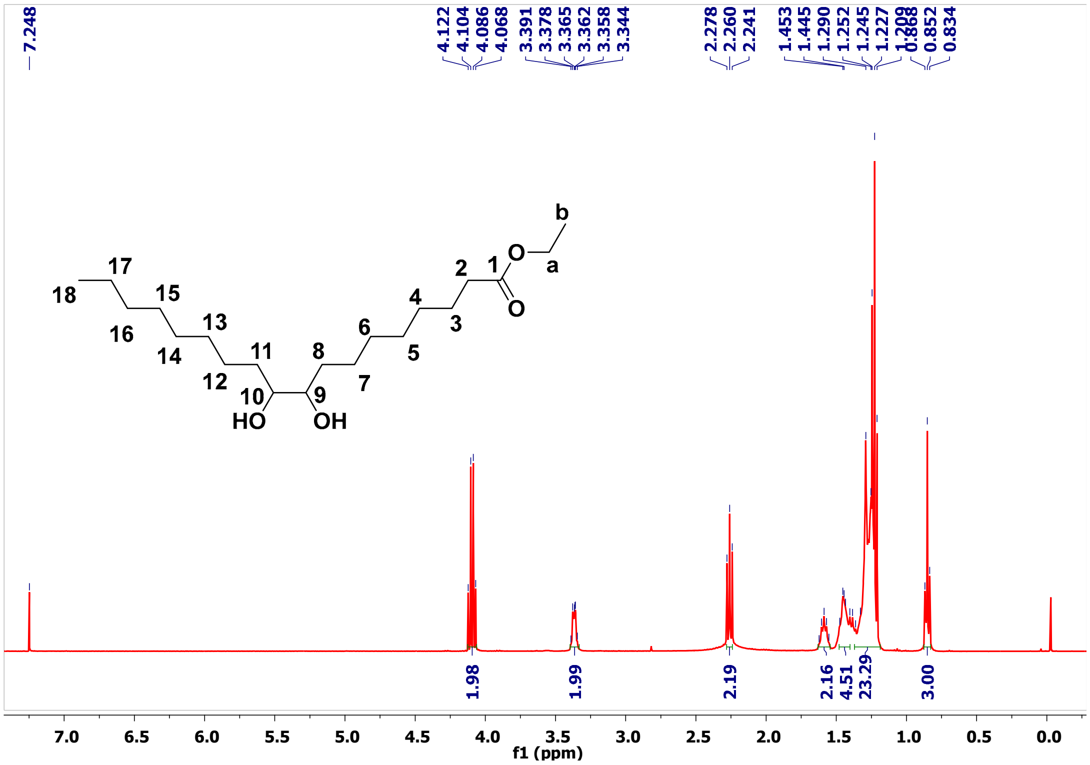

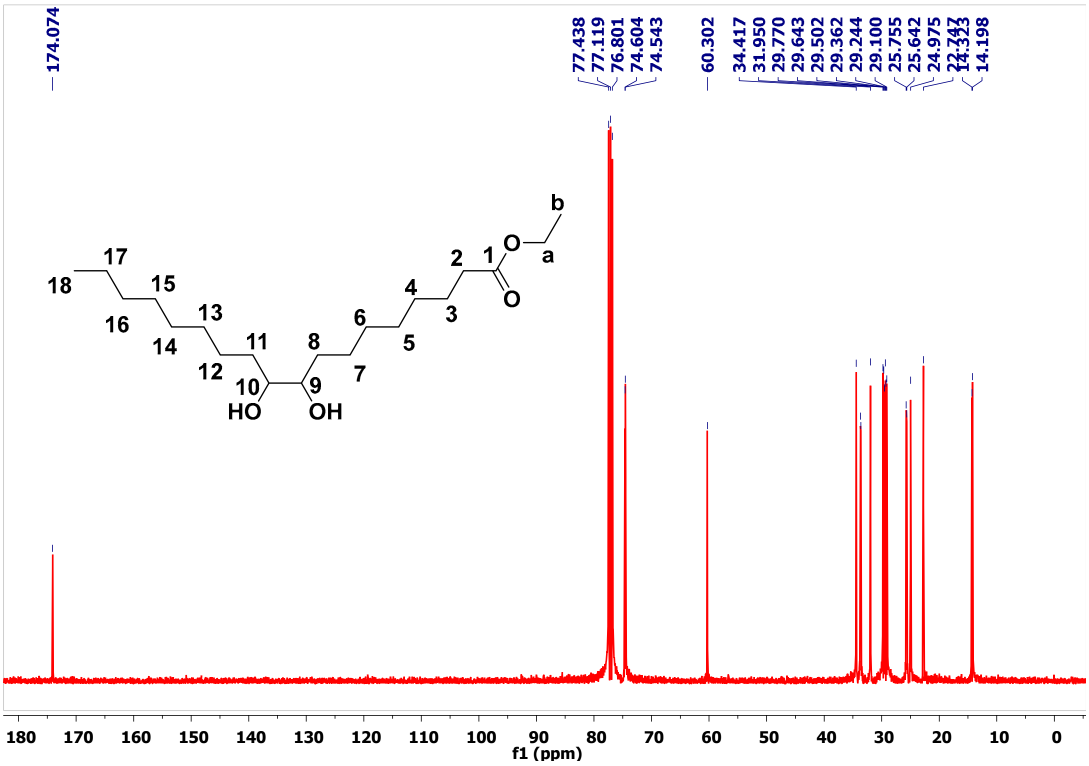

Figure S3. ^{1}H & ^{13}C NMR spectra of compound **4** in $CDCl_3$.

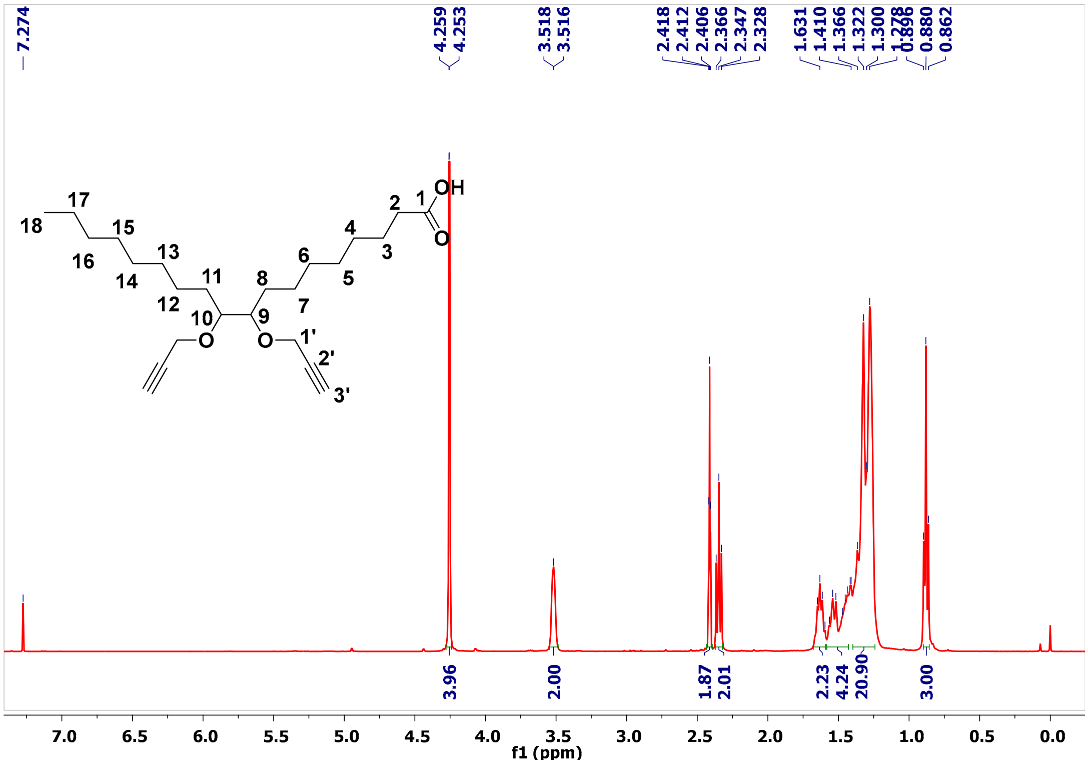

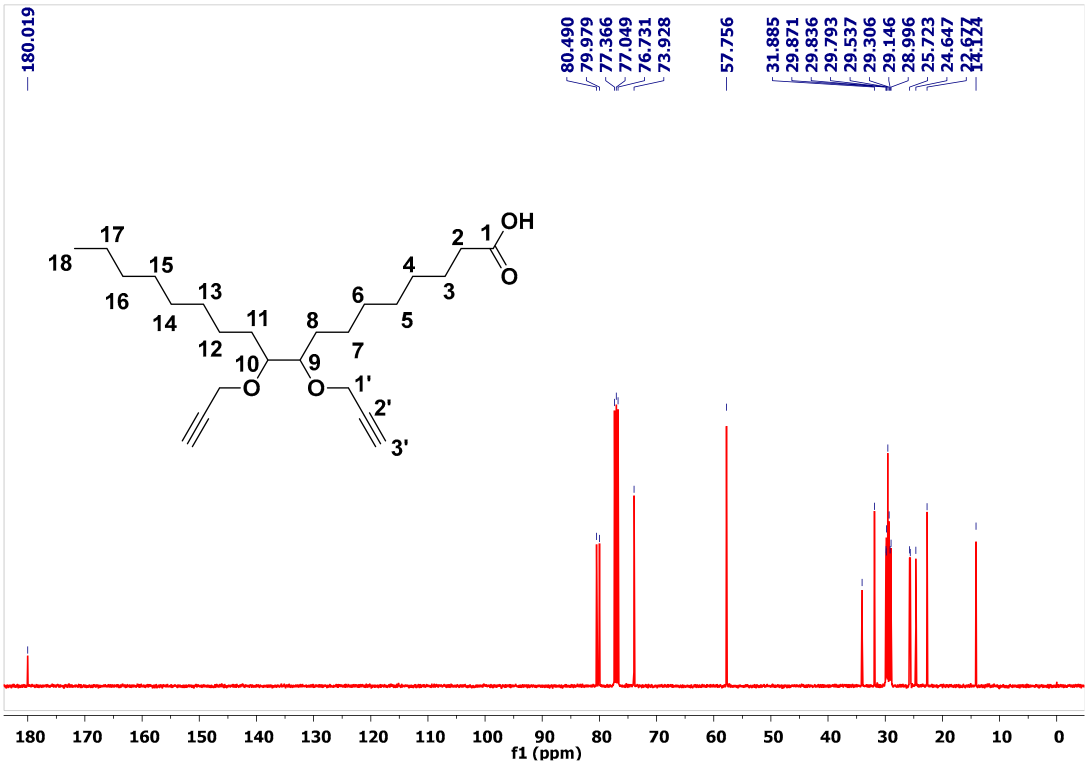

Figure S4. ^{1}H & ^{13}C NMR spectra of compound **5** in $CDCl_3$.

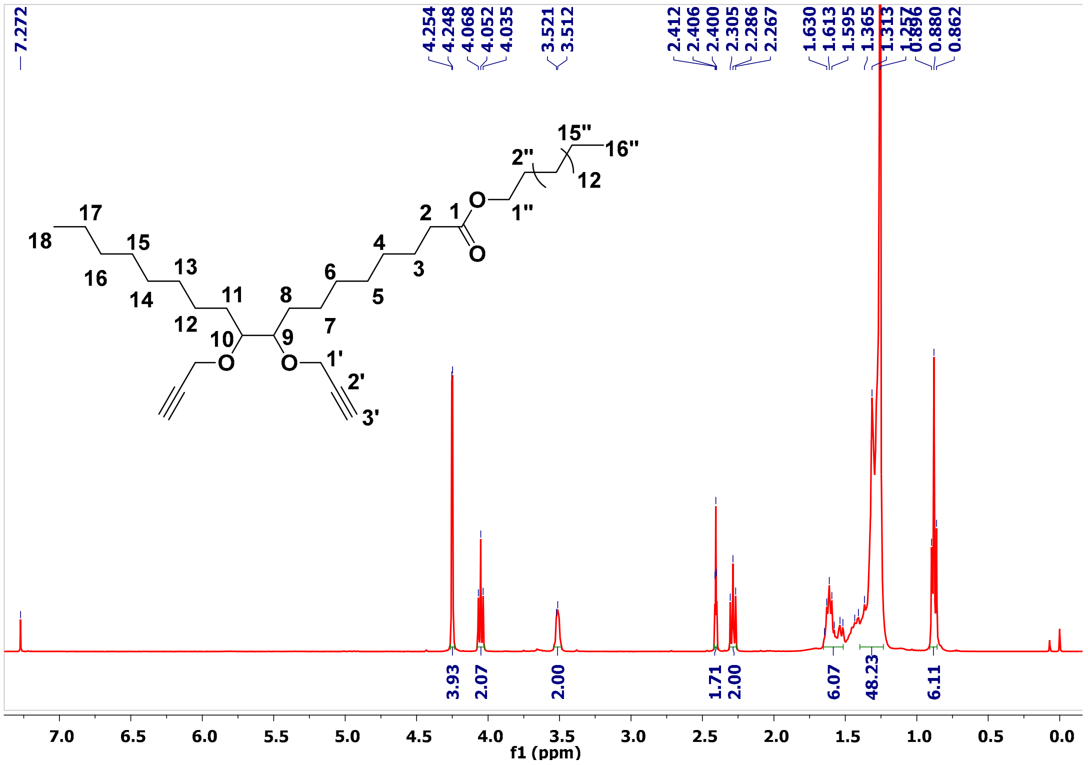

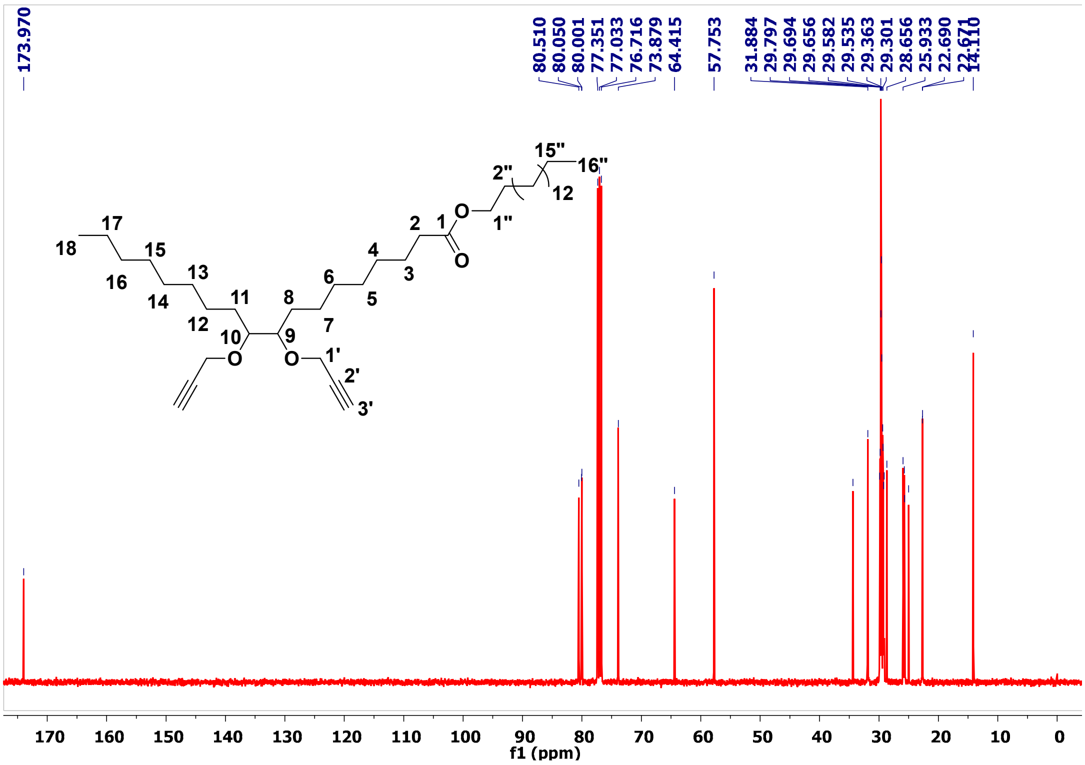

Figure S5. ^{1}H & ^{13}C spectra of compound **6** in $CDCl_3$.

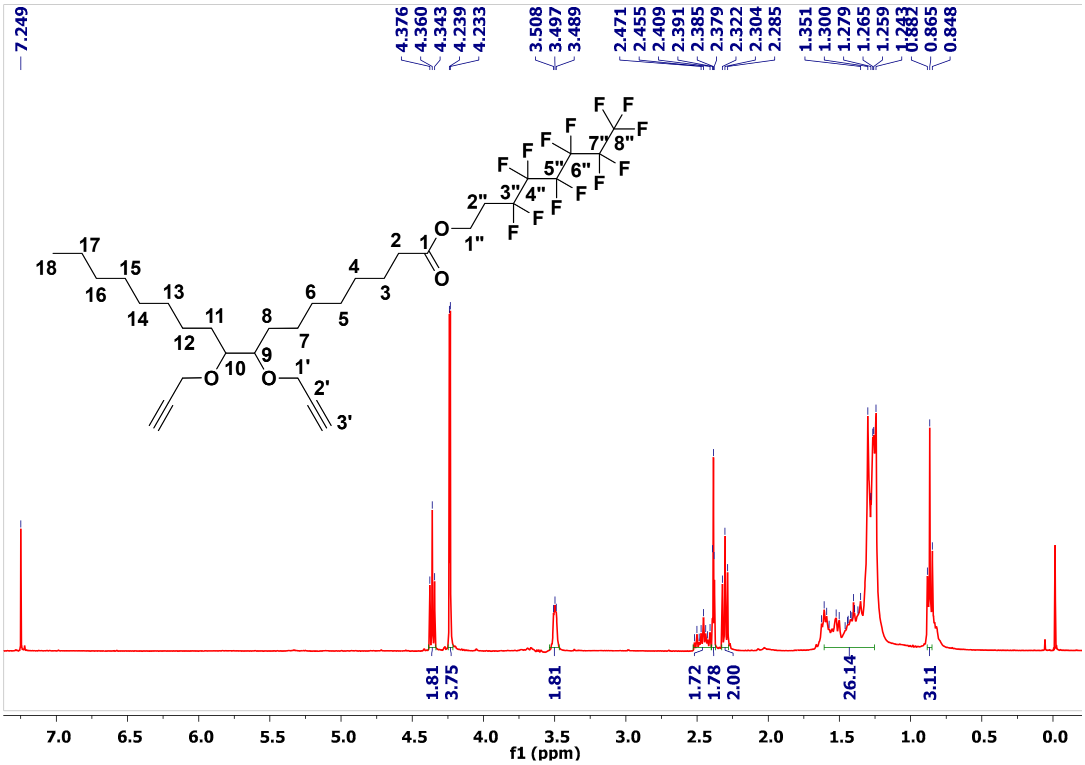

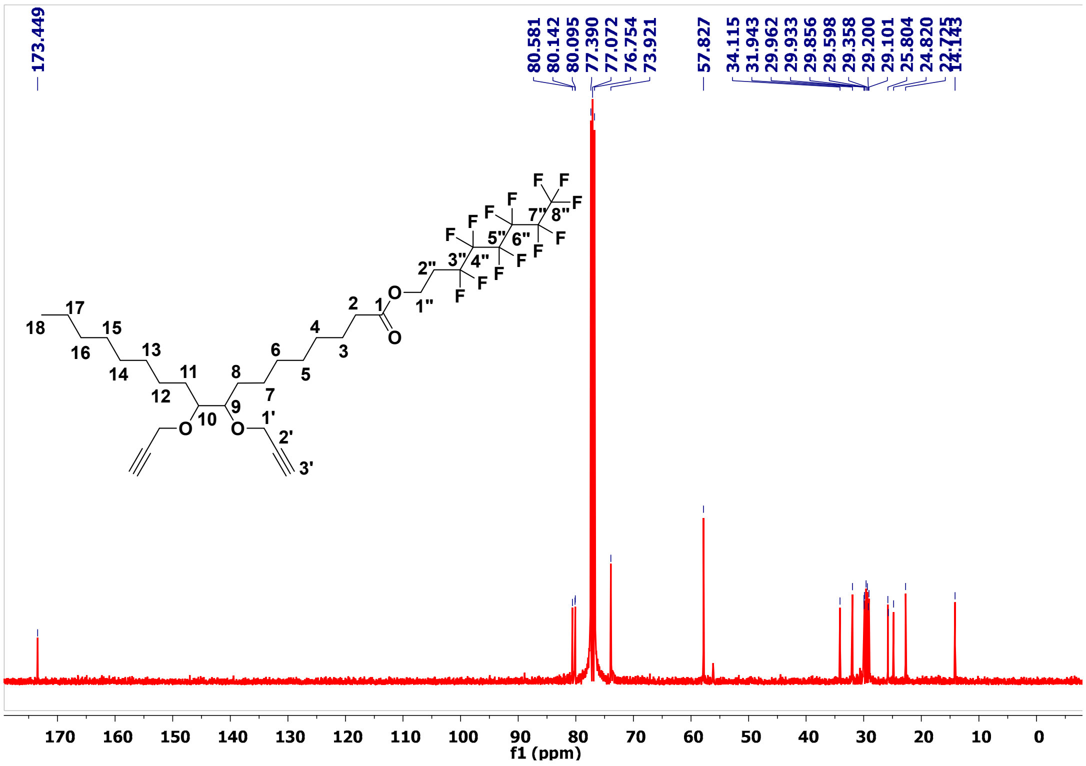

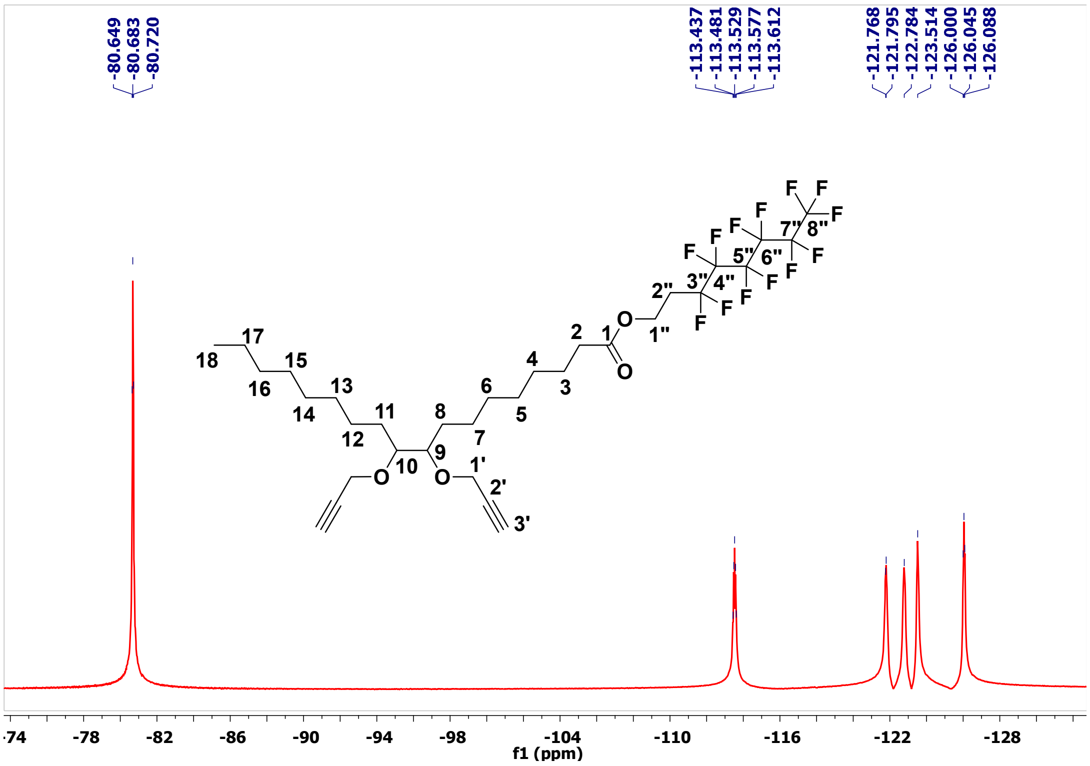

Figure S6. ^{1}H, ^{13}C & ^{19}F NMR spectra of compound **7** in $CDCl_3$.

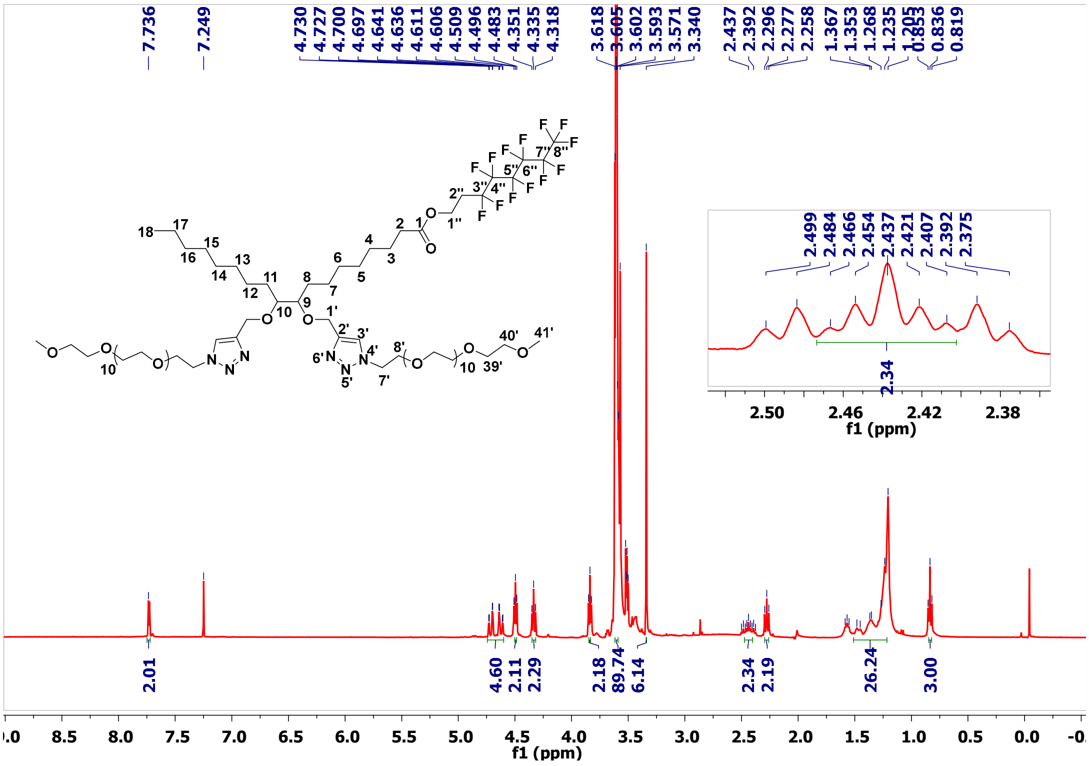
7.736
7.249
4.730 4.727 4.700 4.697 4.641 4.636 4.611 4.606 4.509 4.496 4.483 4.351 4.335 4.318
3.618 3.605 3.602 3.593 3.571 3.340
2.437 2.392 2.296 2.277 2.258
1.367 1.353 1.268 1.235 1.205 0.853 0.836 0.819
2.499 2.484 2.466 2.454 2.437 2.421 2.407 2.392 2.375
2.34
f1 (ppm)
2.01 4.60 2.11 2.29 2.18 89.74 6.14 2.34 2.19 26.24 3.00
f1 (ppm)

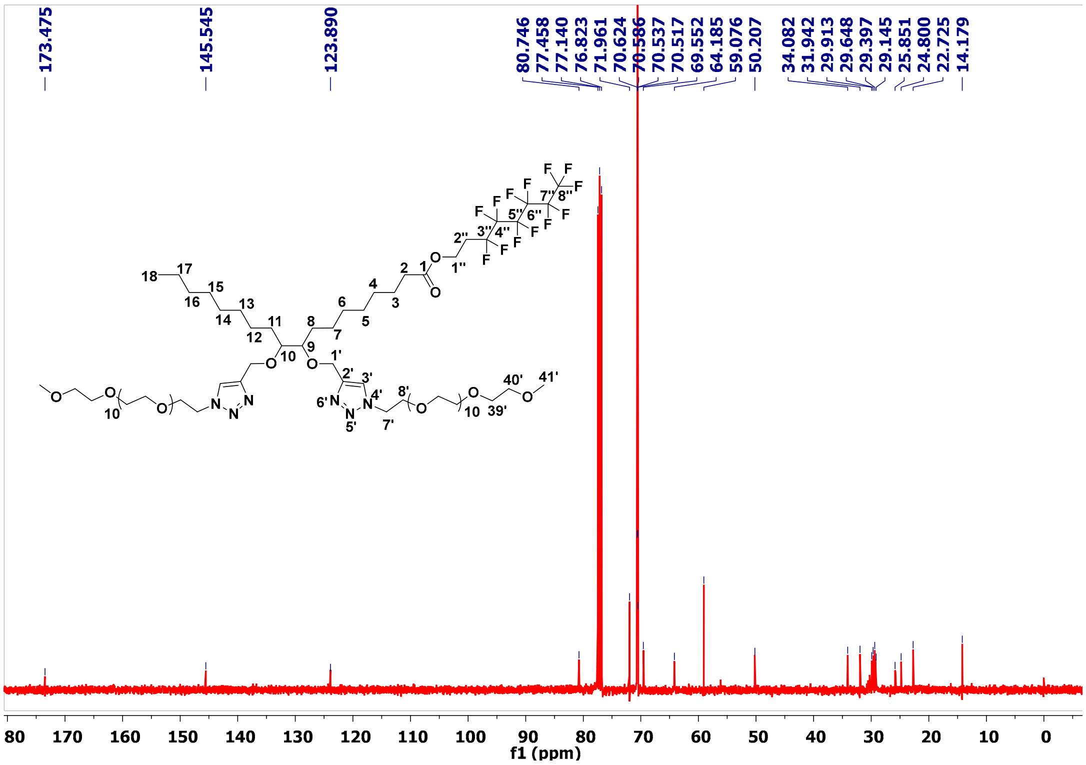
173.475
145.545
123.890
80.746 77.458 77.140 76.823 71.961 70.624 70.586 70.537 70.517 69.552 64.185 59.076 50.207
34.082 31.942 29.913 29.648 29.397 29.145 25.851 24.800 22.725 14.179
f1 (ppm)

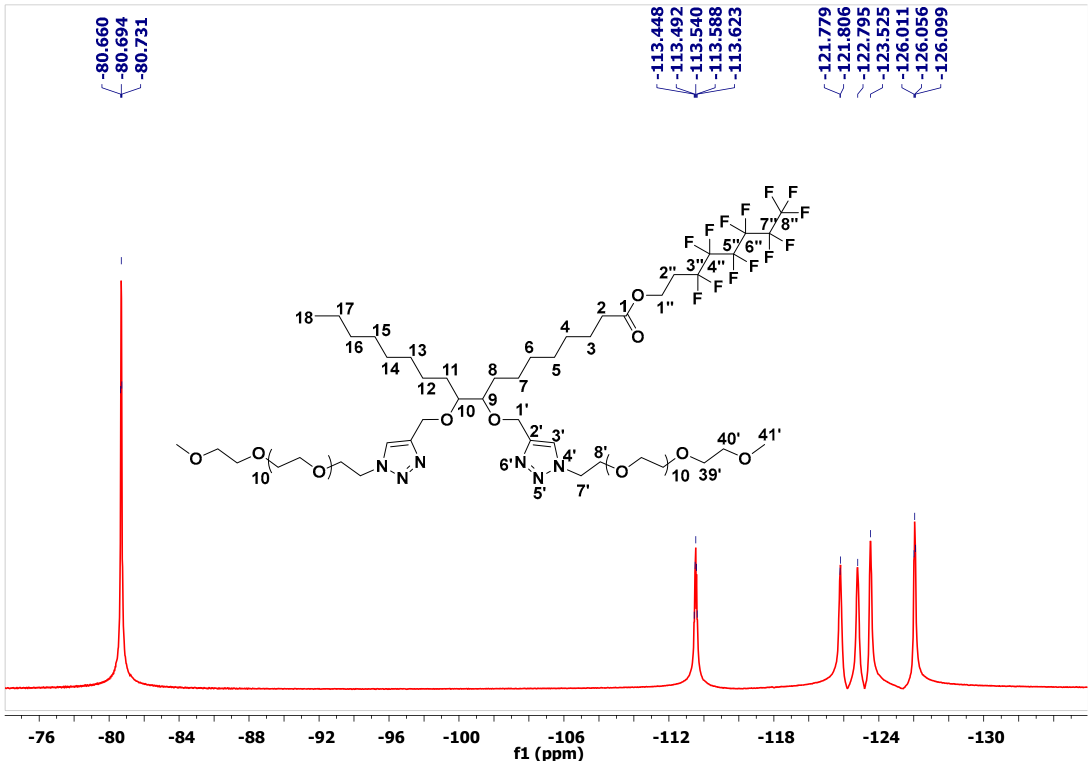

Figure S7. ^{1}H, ^{13}C & ^{19}F NMR spectra of **18** in $CDCl_3$.

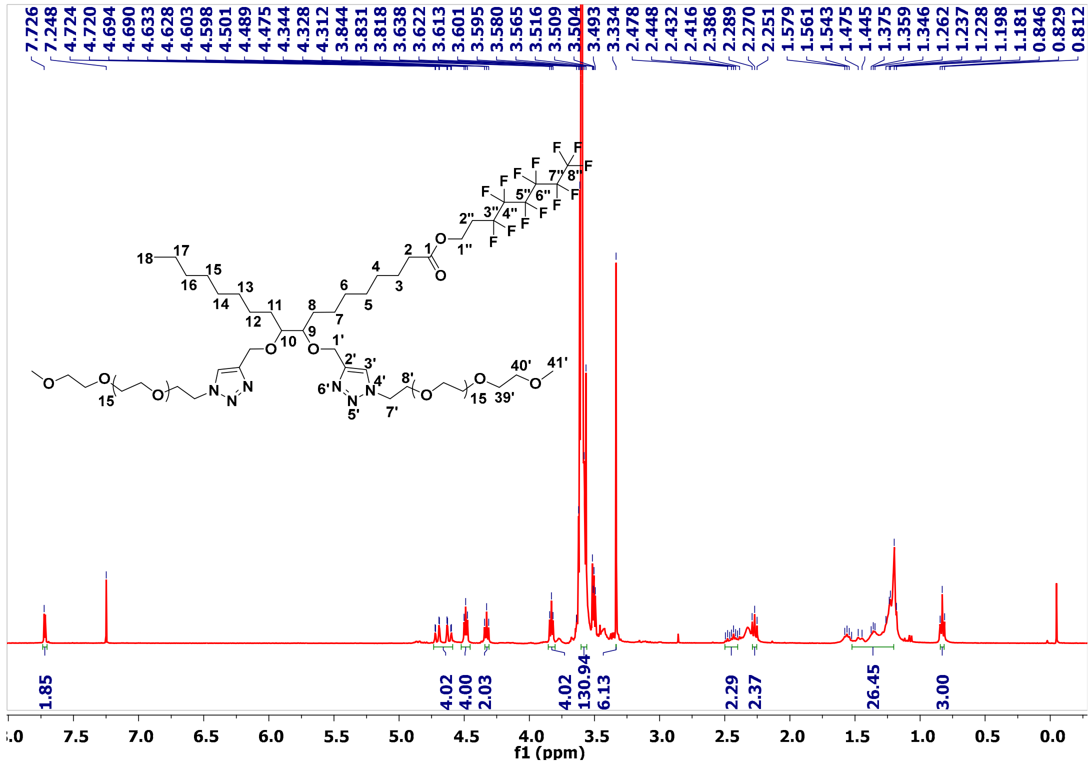

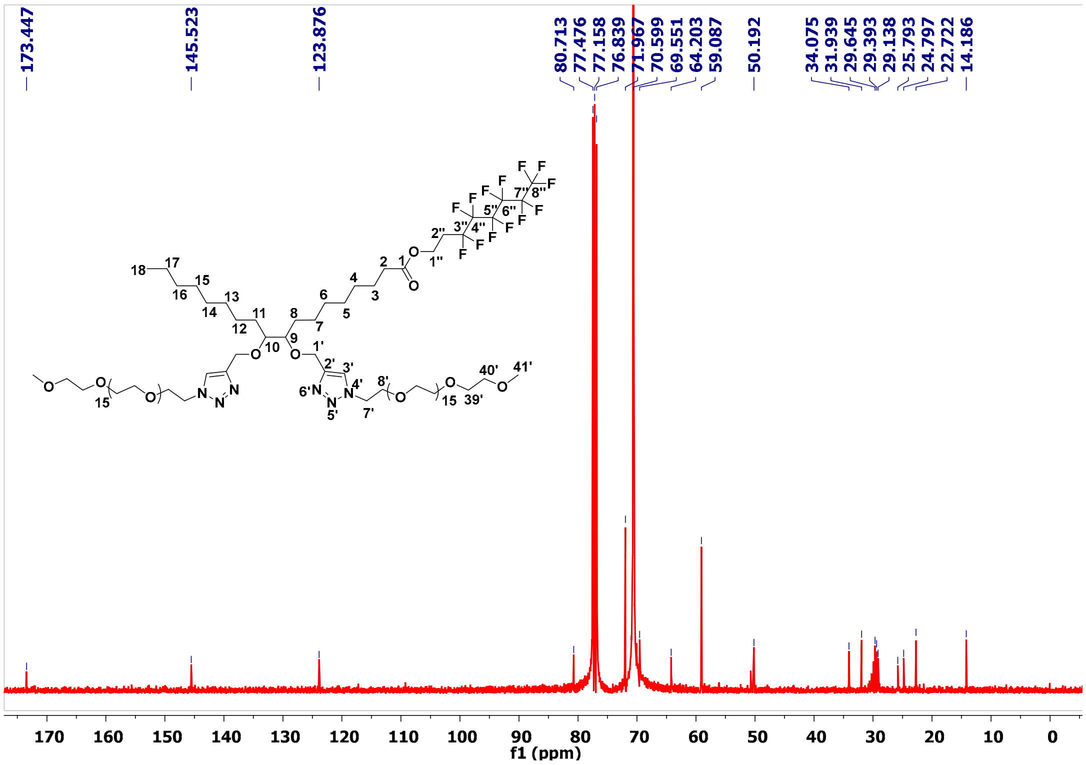

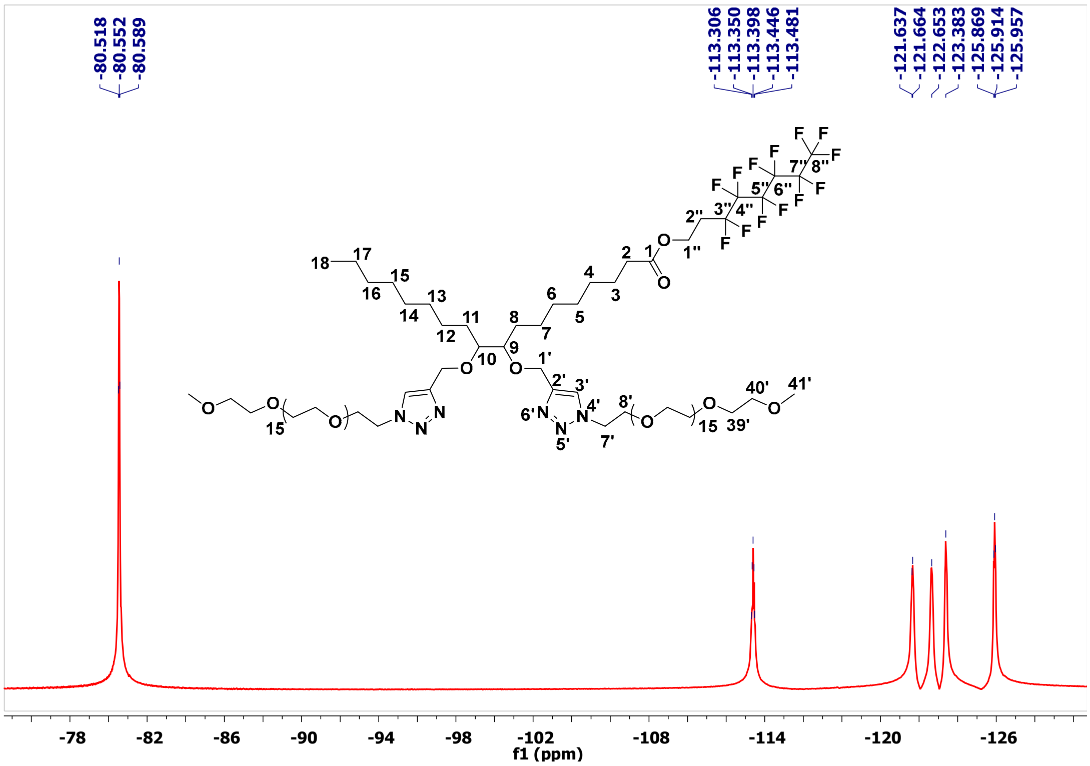

Figure S8. ^{1}H,^{13}C & ^{19}F NMR spectra of **19** in $CDCl_3$.

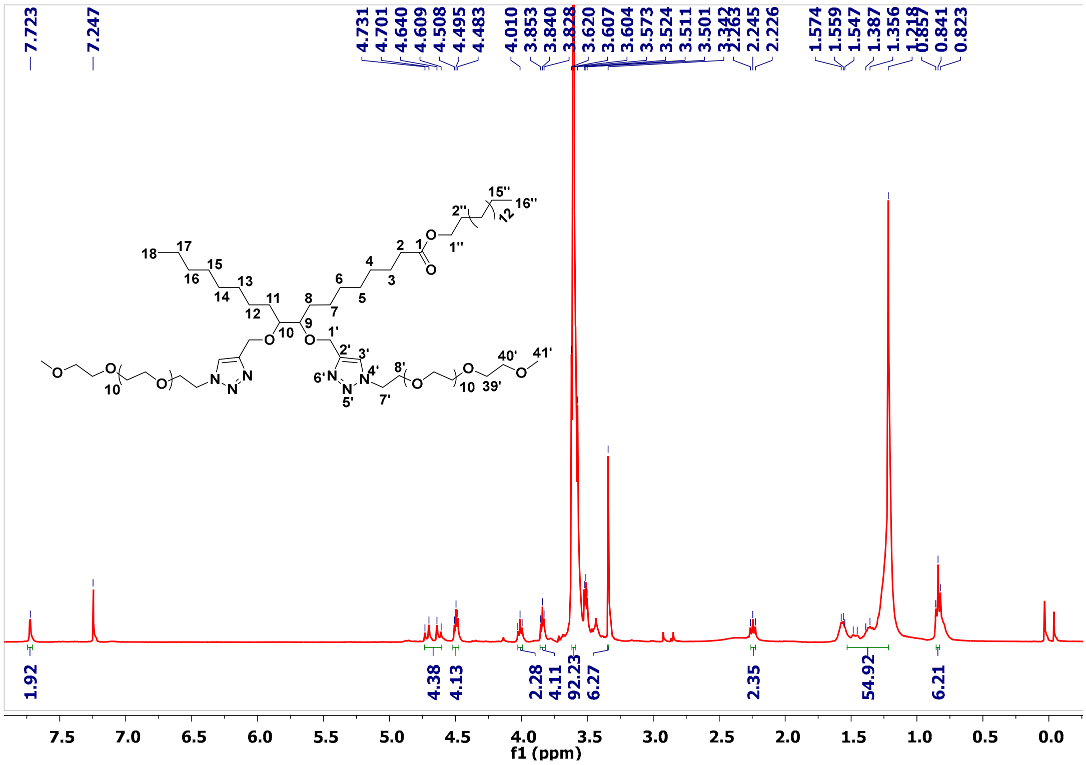

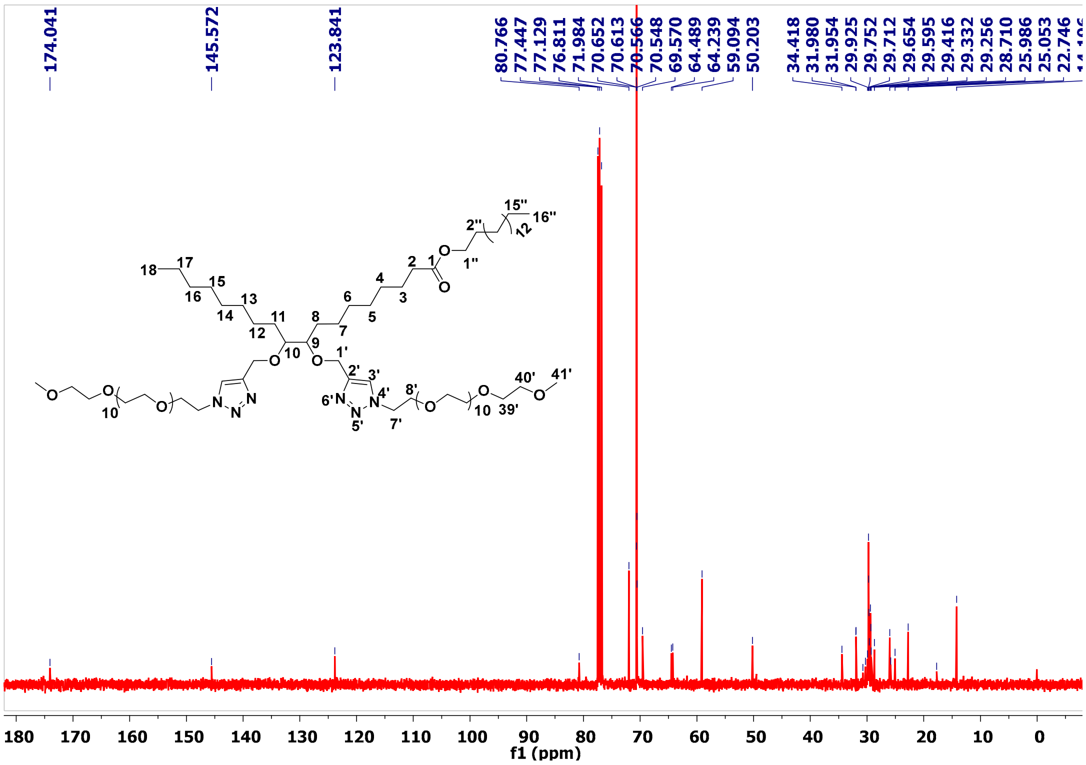

Figure S9. ^{1}H & ^{13}CNMR spectra of **20** in $CDCl_3$.

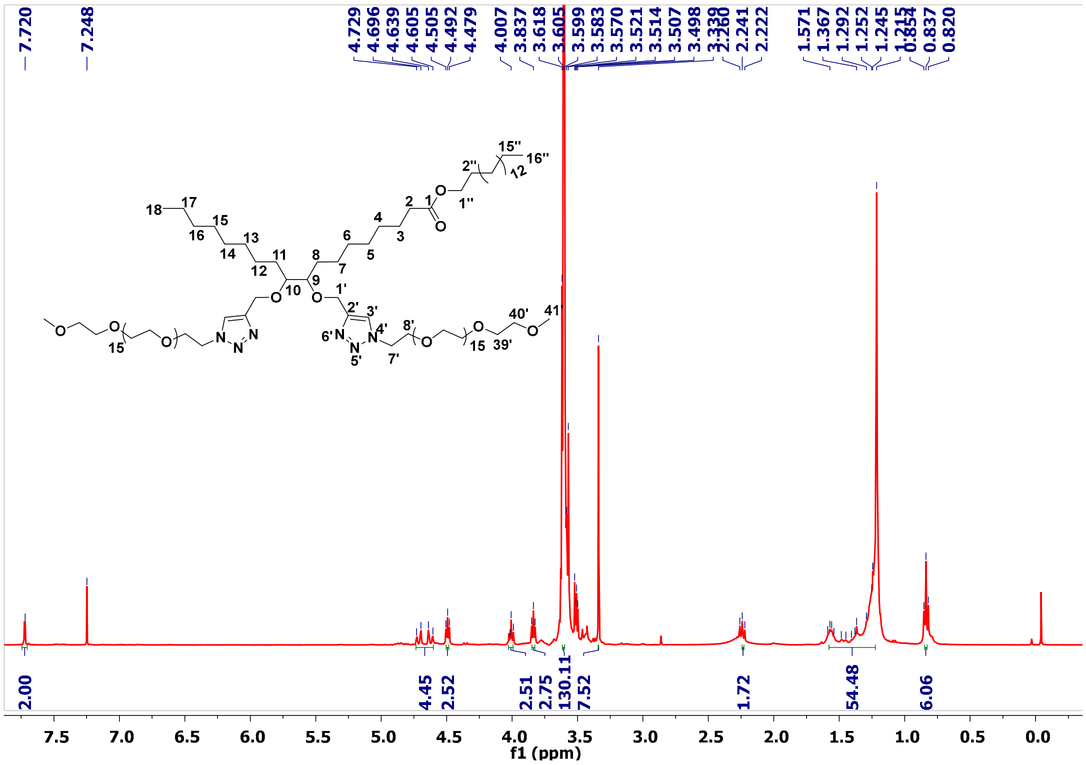

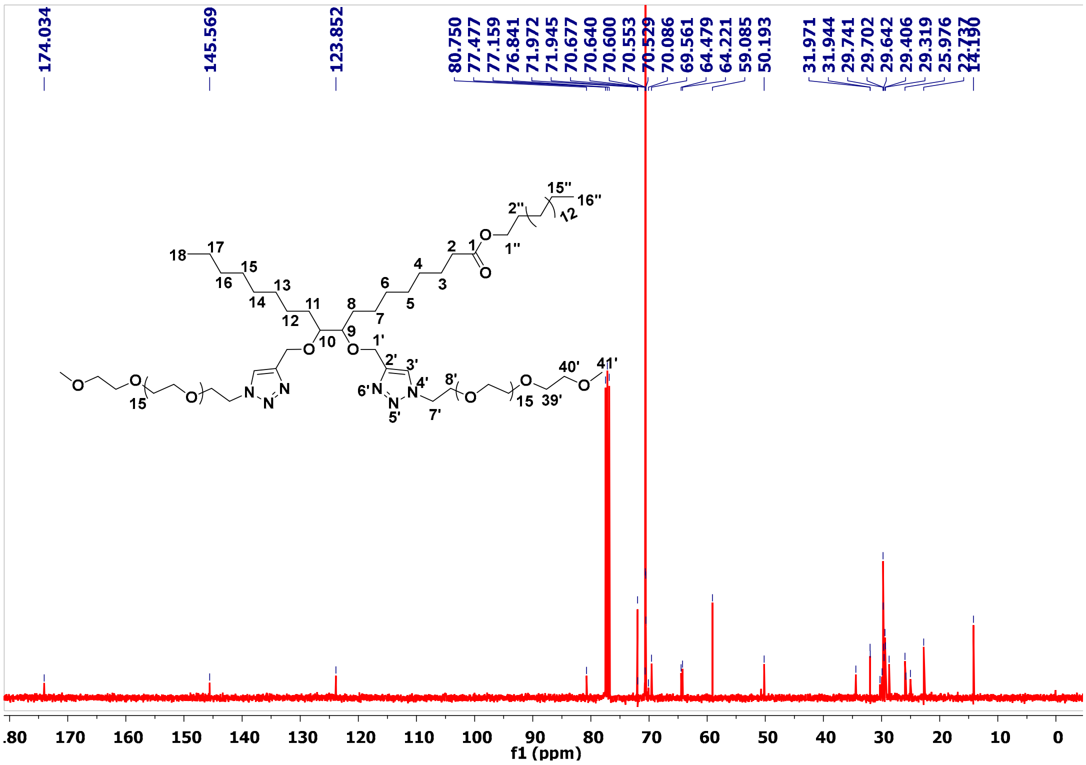

Figure S10. ^{1}H & ^{13}C NMR spectra of **21** in $CDCl_3$.

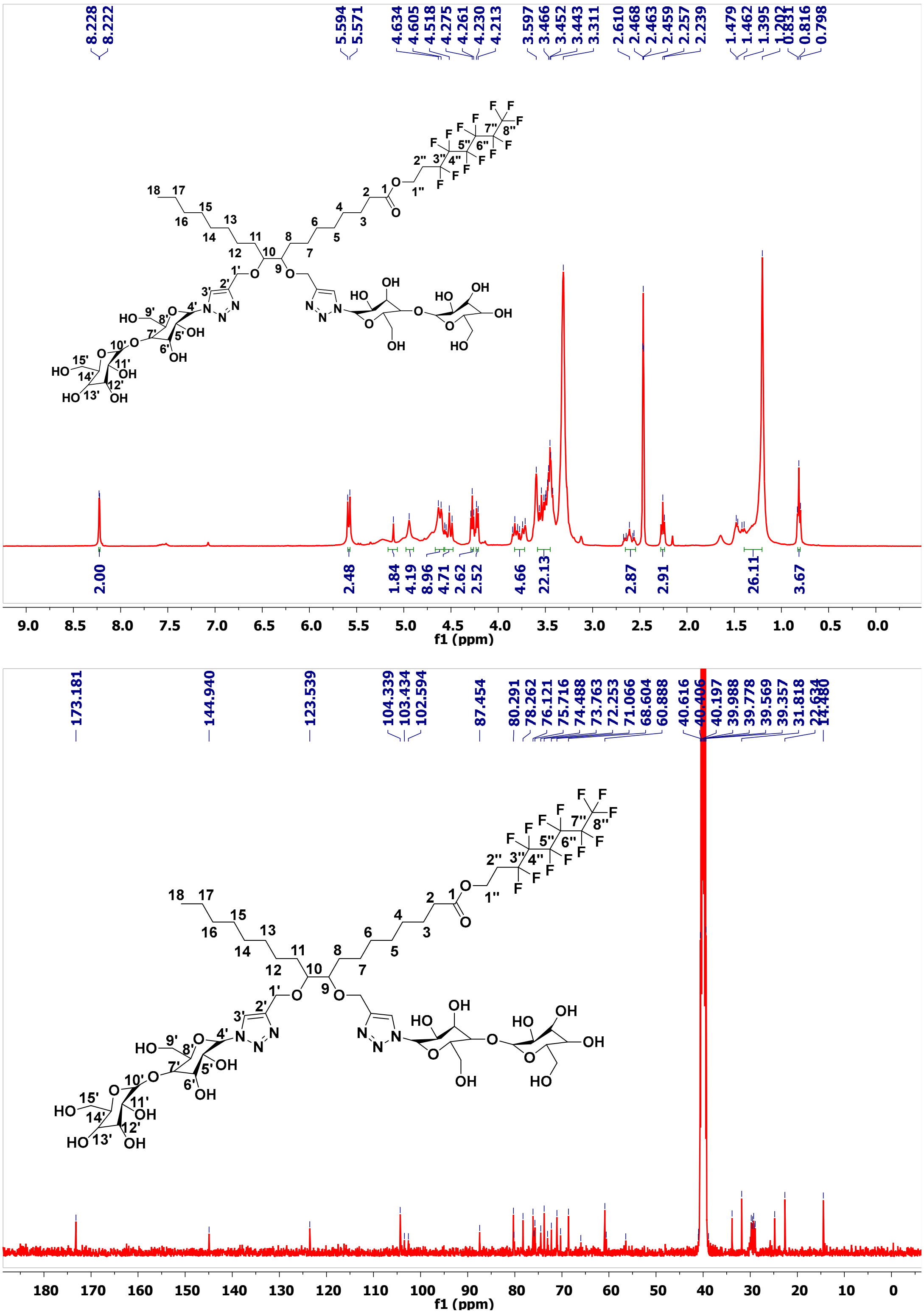

Figure S11. ^{1}H, ^{13}C & ^{19}F NMR spectra of **22** in DMSO-*d*6.

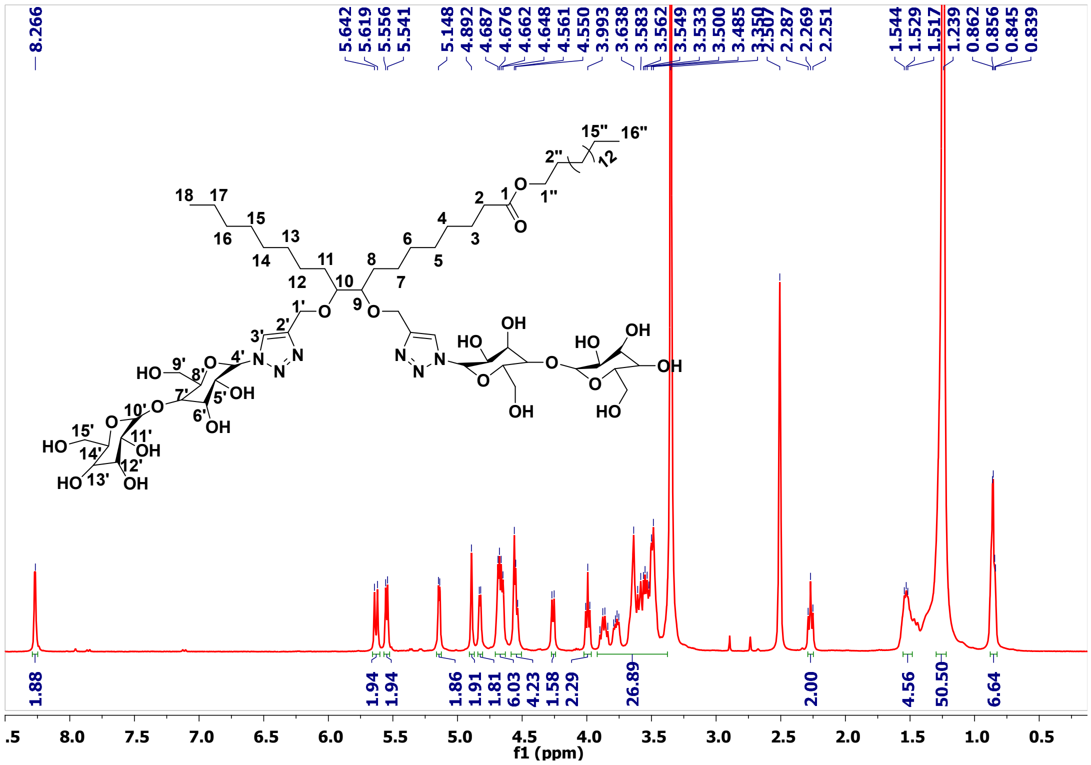

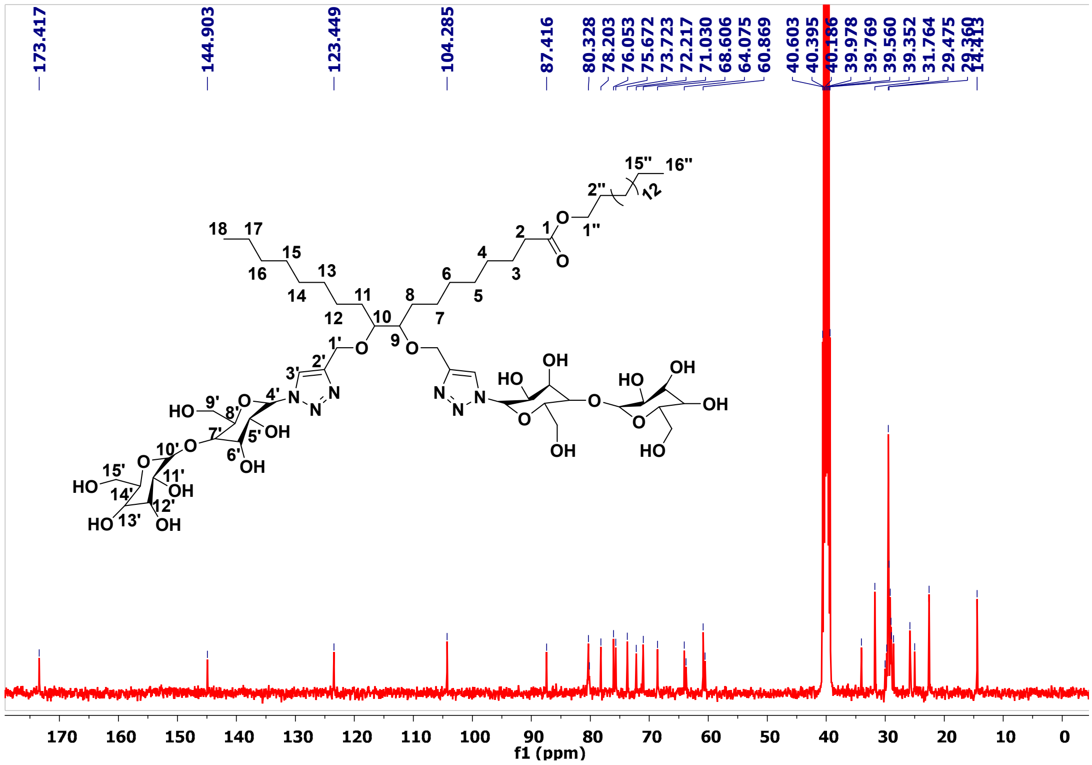

Figure S12. ^{1}H & ^{13}C NMR spectra of **23** in DMSO-d_6.

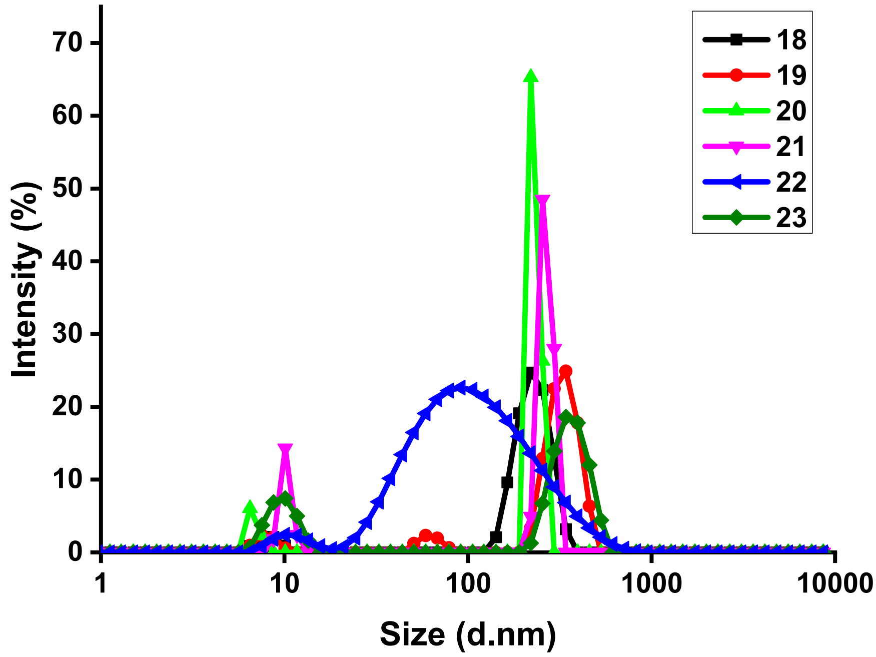

Figure S13. DLS intensity distribution plots of amphiphiles **(18-23)**.

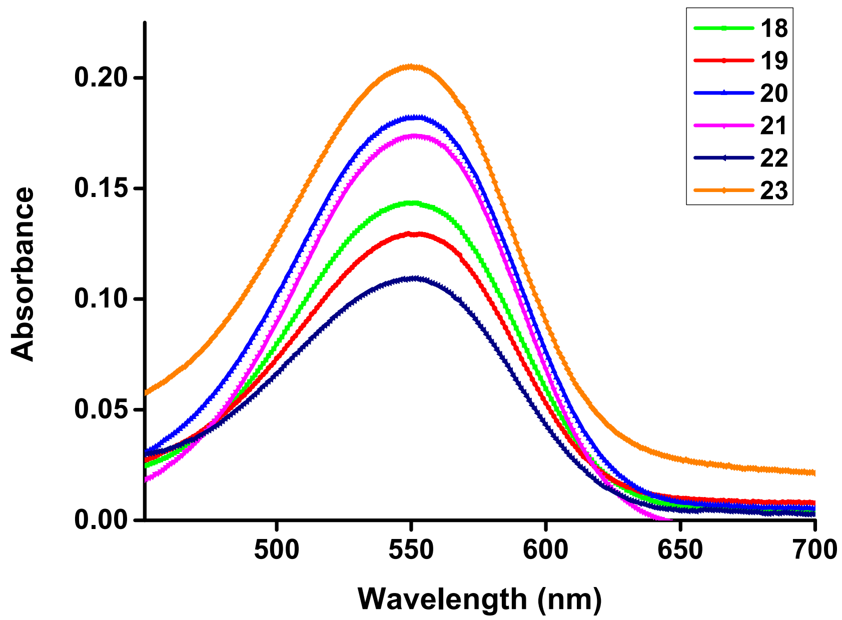

Figure S14. Quantification of Nile red encapsulation in amphiphiles **(18-23)** using UV absorption spectra.

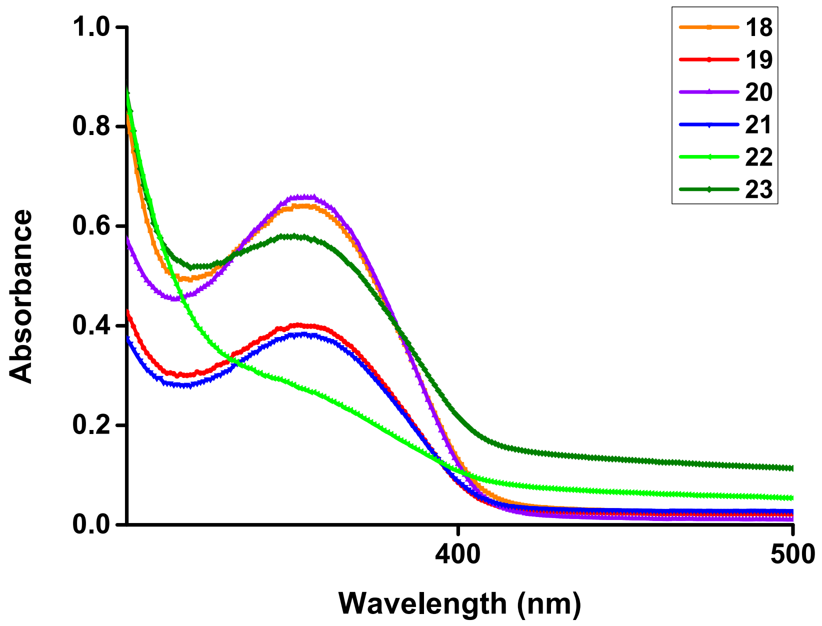

Figure S15. Quantification of nimodipine encapsulation in amphiphiles **(18-23)** using UV absorption spectra.

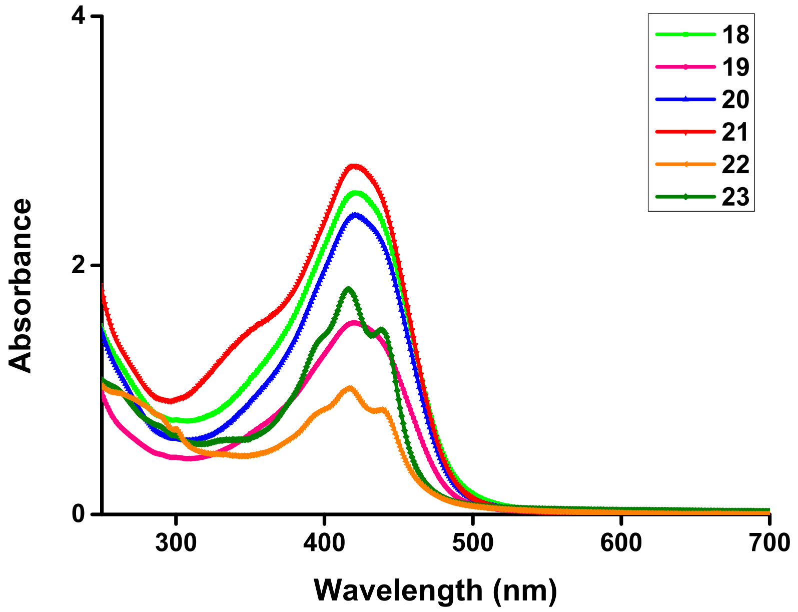

Figure S16. Quantification of curcumin encapsulation in amphiphiles **(18-23)** using UV absorption spectra.

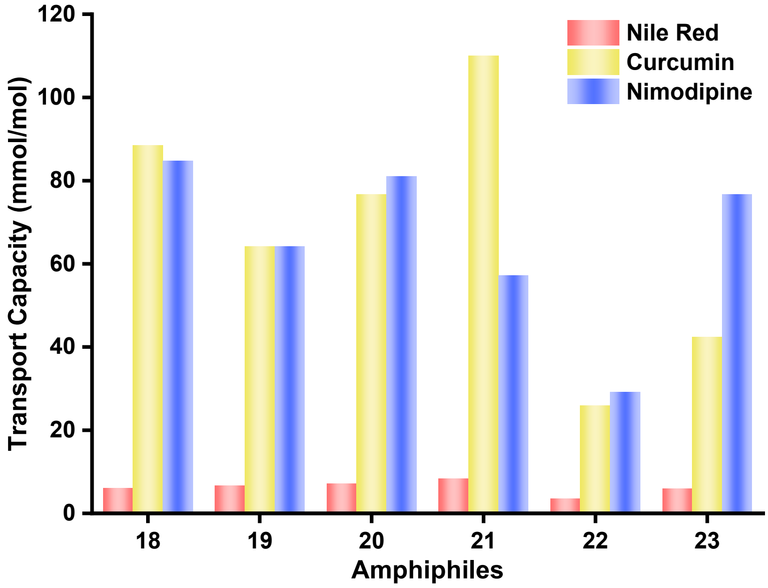

Figure S17. Transport capacity of nanocarriers (**18-23**) for Nile red, nimodipine and curcumin.

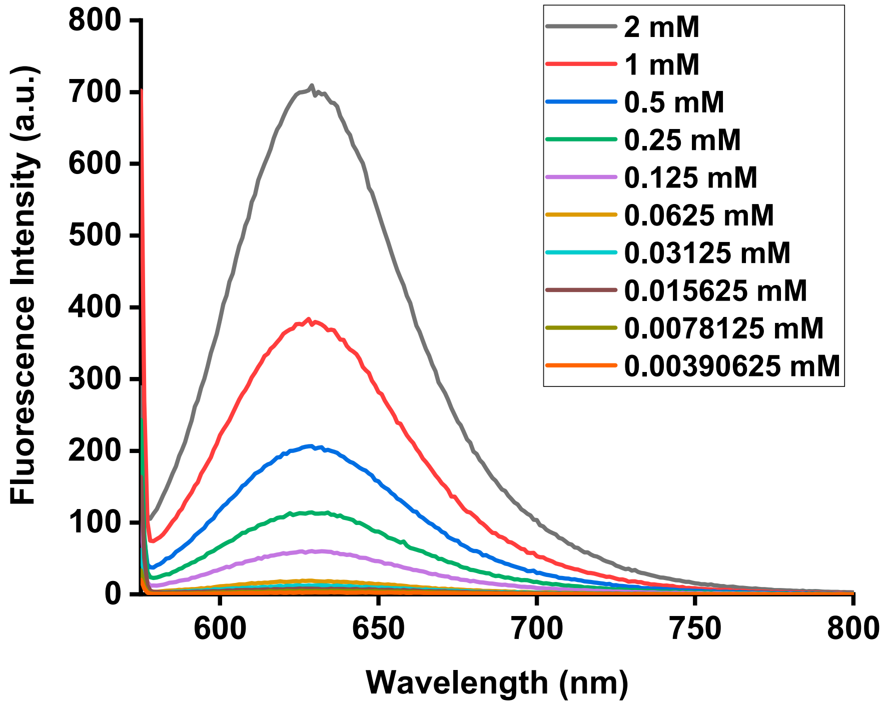

Figure S18. Fluorescence emission spectra of amphiphile **22** at different concentrations.

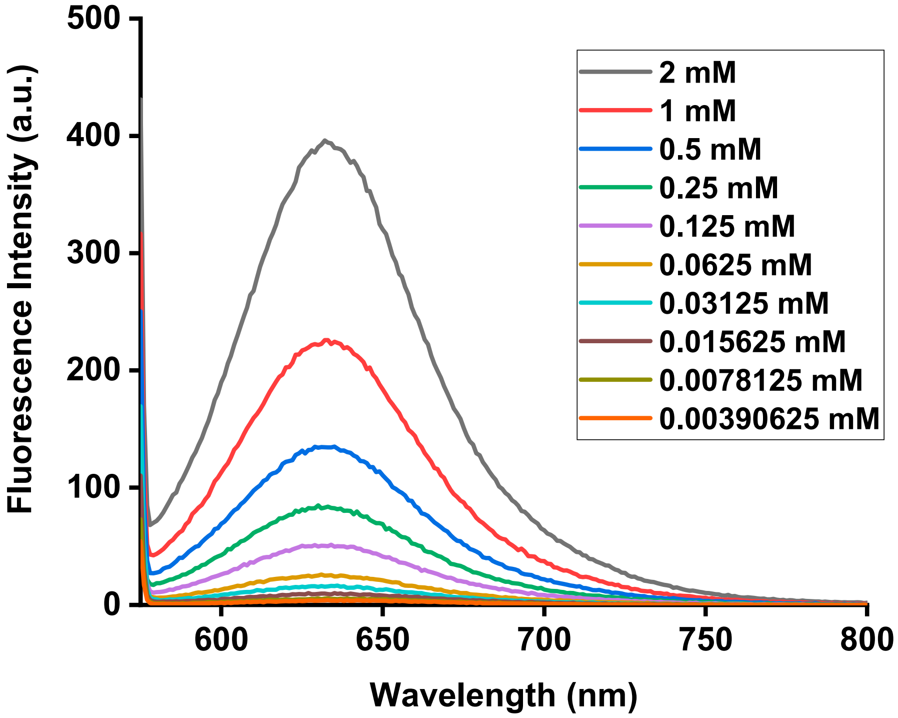

Figure S19. Fluorescence emission spectra of amphiphile **23** at different concentrations.

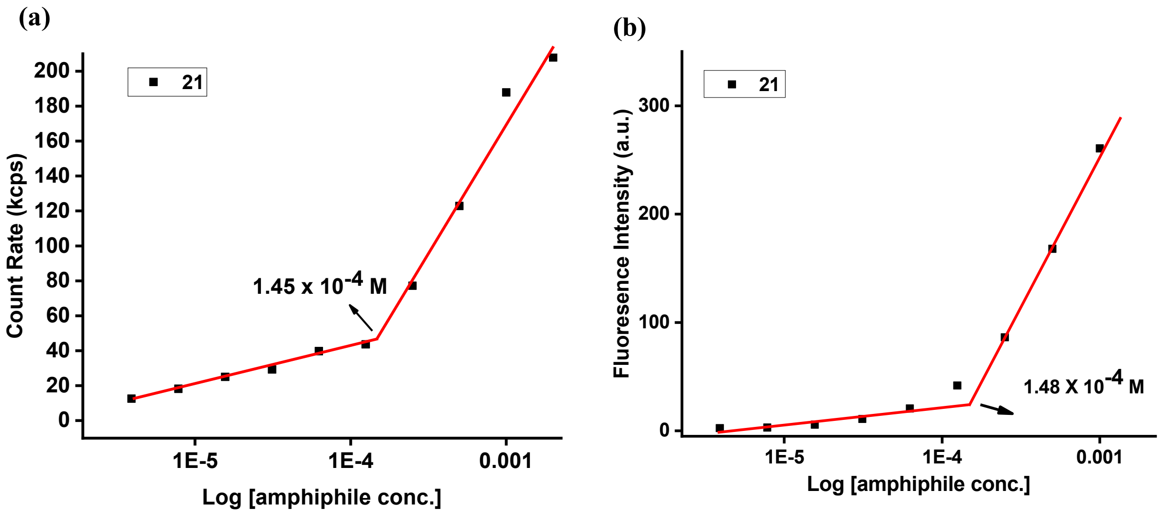

Figure S20. Comparative determination of the critical aggregation concentration (CAC) of amphiphile 21 using two independent methods: **(a)** DLS count-rate method, based on the plot of count rate (kcps) versus log amphiphile concentration, and **(b)** Nile Red fluorescence method, based on the plot of fluorescence intensity versus log amphiphile concentration.

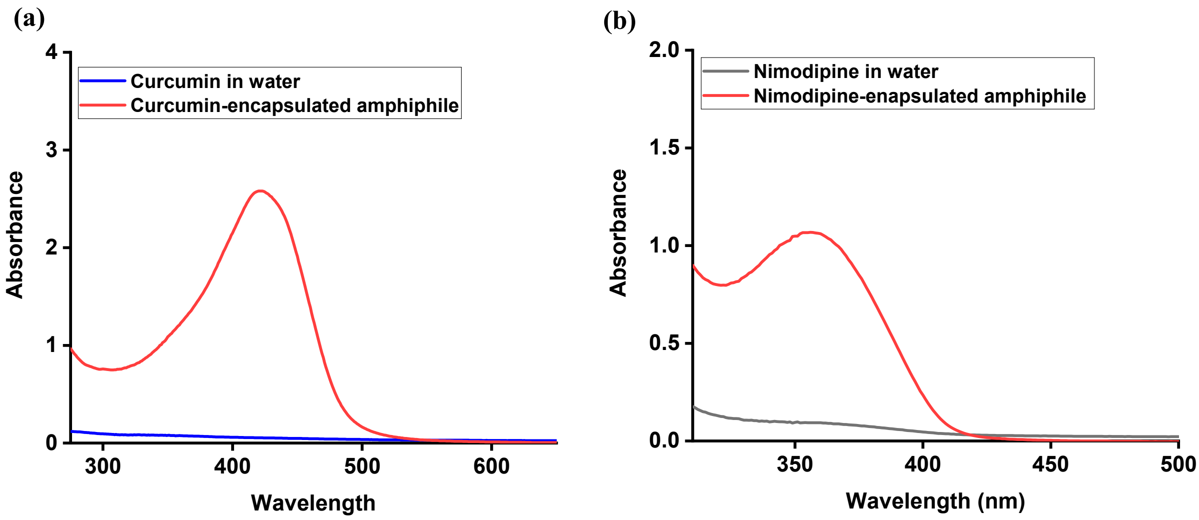

Figure S21. UV–Vis absorption spectra of **(a)** curcumin and **(b)** nimodipine in aqueous medium in the absence and presence of the synthesized amphiphile. The increased absorbance in the presence of the amphiphile indicates an enhanced apparent amount of the respective compound solubilized in the aqueous medium under otherwise identical experimental conditions.

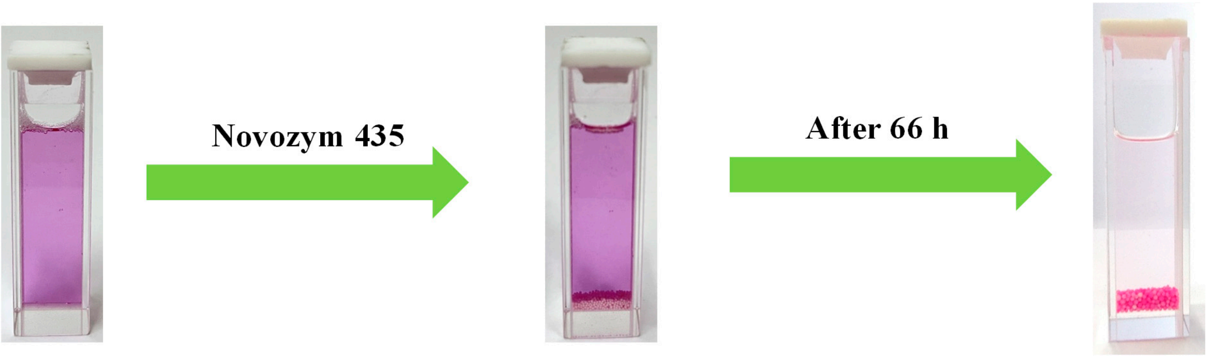

Figure S22. Time-dependent release of Nile red in the presence of enzyme.
